# Host Control of Malaria Infections: Constraints on Immune and Erythropoeitic Response Kinetics

**DOI:** 10.1371/journal.pcbi.1000149

**Published:** 2008-08-22

**Authors:** Philip G. McQueen, F. Ellis McKenzie

**Affiliations:** 1 Mathematical and Statistical Computing Laboratory, Division of Computational Bioscience, Center for Information Technology, National Institutes of Health, Bethesda, Maryland, United States of America; 2 Fogarty International Center, National Institutes of Health, Bethesda, Maryland, United States of America; Utrecht University, Netherlands

## Abstract

The two main agents of human malaria, *Plasmodium vivax* and *Plasmodium falciparum*, can induce severe anemia and provoke strong, complex immune reactions. Which dynamical behaviors of host immune and erythropoietic responses would foster control of infection, and which would lead to runaway parasitemia and/or severe anemia? To answer these questions, we developed differential equation models of interacting parasite and red blood cell (RBC) populations modulated by host immune and erythropoietic responses. The model immune responses incorporate both a rapidly responding innate component and a slower-responding, long-term antibody component, with several parasite developmental stages considered as targets for each type of immune response. We found that simulated infections with the highest parasitemia tended to be those with ineffective innate immunity even if antibodies were present. We also compared infections with dyserythropoiesis (reduced RBC production during infection) to those with compensatory erythropoiesis (boosted RBC production) or a fixed basal RBC production rate. Dyserythropoiesis tended to reduce parasitemia slightly but at a cost to the host of aggravating anemia. On the other hand, compensatory erythropoiesis tended to reduce the severity of anemia but with enhanced parasitemia if the innate response was ineffective. For both parasite species, sharp transitions between the schizont and the merozoite stages of development (i.e., with standard deviation in intra-RBC development time ≤2.4 h) were associated with lower parasitemia and less severe anemia. Thus tight synchronization in asexual parasite development might help control parasitemia. Finally, our simulations suggest that *P. vivax* can induce severe anemia as readily as *P. falciparum* for the same type of immune response, though *P. vivax* attacks a much smaller subset of RBCs. Since most *P. vivax* infections are nonlethal (if debilitating) clinically, this suggests that *P. falciparum* adaptations for countering or evading immune responses are more effective than those of *P. vivax*.

## Author Summary

Of the four *Plasmodium* species that parasitize human erythrocytes and induce malaria, *Plasmodium falciparum* and *Plasmodium vivax* cause most of the public health burden. *P. falciparum* infection is typically characterized as “malignant” (due to severe, sometimes lethal consequences, particularly in immune-naïve individuals), and *P. vivax* malaria as (relatively) “benign.” Using the power of a Beowulf cluster, we tested hypotheses about host control of malaria by simulating ∼8.4×10^4^ combinations of parasite species, host immune response, and erythropoietic response to infection. We tailored the models to specific details of the life cycles of the two species, which invade different subclasses of red blood cells. Our results challenge some standard assumptions. For example, we show that tight synchronization of the asexual reproduction of malaria parasites may actually benefit the host by reducing parasitemia. We also demonstrate that properties of host immunity or erythropoiesis that contribute to high parasitemia and severe anemia in *P. falciparum* malaria would do so in *P. vivax* infection as well, in line with recent reports indicating that *P. vivax* can indeed cause “malignant” illness in some patients. This suggests that *P. falciparum* is more effective overall at immune evasion or suppression than *P. vivax*.

## Introduction

The parasites that cause human malaria are inoculated by an *Anopheles* mosquito and initially multiply in the liver. After about a week, a primary wave of merozoite forms enters the bloodstream, invades RBCs (within seconds), and continues the asexual cycle of multiplication, developing into the schizont forms that burst and release more merozoites. The pathology of malaria is due to this asexual blood- stage cycle (reviewed in [1],[2]). The sexual forms transmissible to mosquitoes (gametocytes) appear over time, but in much smaller numbers than the asexual forms. The two parasite species that cause the vast majority of human cases, *Plasmodium vivax* and *Plasmodium falciparum*, can induce severe anemia; with *P. vivax* especially, the anemia can appear far out of proportion to the percentage of RBCs infected [3].

Both the innate and adaptive arms of the human immune system mount responses to infections with both species (reviewed in [4],[5]). High fevers are a classic feature of infections. During *P. vivax* infections near-periodic episodes of fever (“paroxysms”) are associated with high levels of tumor necrosis factor α (TNF-α) and other cytokines associated with innate immunity [6]–[8]. Strong cytokine responses also occur in *P. falciparum* infection, though the timing of paroxysms tends to be irregular [9]–[11]. These fever paroxysms are associated with the synchronized release of merozoites from bursting schizonts. This synchronization has been the subject of considerable experimental and theoretical work. It is possible that febrile temperatures induce synchronization by differentially influencing development rates of different parasite stages [12] and that immune responses [13],[14] as well as the host's melatonin release cycle [15]–[17] contribute to this phenomenon. But it is not yet clear whether synchronization helps parasites, perhaps in the way a sudden overwhelming abundance of prey may overwhelm a predator's capacity, or hinders them. Malaria parasites have certainly evolved mechanisms of immune evasion; however, *P. falciparum* exhibits antigenic variation [18], adheres to vascular endothelium in response to fever [19], and produces prostaglandins which probably modulate host TNF-α production [20]. This species also manages to keep the membrane of infected RBCs deformable during its ring stage (RBCs in the first 24 hours after infection), apparently reducing exposure of ring-stage parasites to clearance by the spleen [21]. *P. vivax* can also evade spleen clearance (reviewed in [22]) and suppress immune responses directed against its liver stage [23].

Clinical investigations suggest that the malaria parasite and host immune response interact with the host erythropoietic system in a complex, dynamic manner (reviewed in [24]). Increased production of TNF-α by the host apparently induces anemia [25]; experimental evidence suggests that hemozoin produced by *P. falciparum* suppresses RBC production [26]. Abnormalities observed in *P. falciparum*-infected patients include suppression of erythroid progenitor cells in bone marrow [27], sequestration of parasites in the marrow [28], decreased iron absorption and hemoglobin synthesis [29], and decreased RBC survival time [30]. Leucocyte infiltration of the marrow and erythroblast degradation by macrophages have been observed in *P. vivax* infections [31]. Phagocytosis of uninfected RBCs has been observed in vitro and in *P. berghei*-infected mice [32], and is suspected in human malaria [33],[34]. *P. chabaudi*-infected mice show enhanced erythropoiesis that can compensate for RBC loss [34]; in humans, elevated levels of erythropoietin are produced in response to *P. falciparum* infection [35]. Overall, the evidence suggests that RBC destruction or ineffective erythropoiesis may thwart erythropoietin-initiated processes that might otherwise compensate for RBC loss, although erythropoietin may have other protective effects [36].

Most fatal malaria infections are due to *P. falciparum*, which can induce cerebral complications as well as severe anemia. *P. vivax* infections are characterized by lower levels of parasitemia, and, though often debilitating, are rarely fatal. *P. falciparum* attacks RBCs of all ages, while *P. vivax* mainly attacks reticulocytes (RBCs<1.5 days old, still showing remnants of nuclei) [37]–[39], and possibly RBCs up to two weeks old [40]. In a previous report, we argued that targeted depletion of the youngest RBCs makes *P. vivax* infection potentially much more dangerous than is commonly appreciated: unchecked growth of a *P. vivax* population would eventually prevent the replacement of older, uninfected RBCs as these senesce and are culled from the circulation [41]. Thus one might expect a strong immune response to *P. vivax*, despite its seemingly lower threat relative to *P. falciparum*. Furthermore, in that model—which did not incorporate an immune response—compensatory boosting of RBC production tended to increase parasitemia, while dyserythropoietic response (reducing RBC production below its basal rate) had the opposite effect.

**Figure 1:**
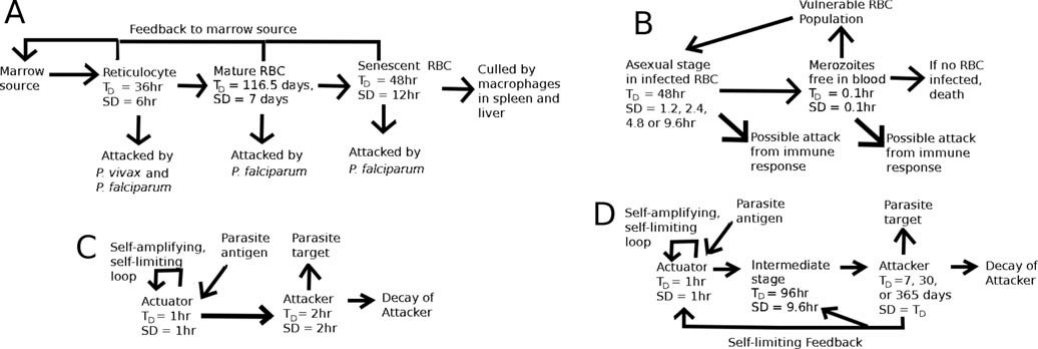
Schematic of the model. (A) The RBC development chain, (B) the parasite development chain, (C) the quick-acting immune response that emulates the innate response, (D) the longer-acting immune response that emulates an antibody response. *T*_D_ = average duration of a stage of development, and SD = standard deviation in the stage duration.

Here we consider compartmentalized ordinary differential equations (CODEs) representing *P. vivax* and *P. falciparum* infections which incorporate a quick-acting innate response and a longer-term acquired antibody response as well as a dynamic erythropoietic system. Figure 1 shows the basic scheme; the details are presented below in the Model section. The innate response emulates aspects of the fever paroxysm response which is the hallmark symptom of malaria. We analyze how these components jointly affect parasite and RBC dynamics. We do not attempt to model the full complexity of the immune response in malaria infections: our aim is to assess potential trade-offs between host and parasite for given characteristics of immune and erythropoietic responses. However, we do consider several choices of targets for both the innate and antibody responses in the model. We assume that bursting schizonts activate the innate response, and that the stage that triggers the antibody response is the same as the stage it targets. (The intraerythrocyte stage terminology is explained in the Model section.) We also consider different values for the time constant of decay for the effective clearance rate of parasites by the antibody response. This time constant is not necessarily the biochemical decay constant of actual antibodies as it also incorporates the possibility of a long lived population of B-cells producing the antibodies. The models also incorporate a nonzero standard deviation in the time parasites develop within RBCs. We show that host erythopoietic response can affect infection outcome even in the presence of sustained immunological action. We show how infection outcome varies with the life stages of parasite targeted by the model immune responses. Furthermore, we show that a tight synchronization of merozoite release does not necessarily help parasite populations evade host immune responses. Most of our simulated infections assume that the host has no pre-existing antibodies or memory B cells for the parasite (and thus is “antibody-naïve”), but for some examples we examine the effects of pre-existing antibodies.

## Results

### Typical Simulations

A measurable quantity that constrains the model parameters is the initial reproduction rate of a parasite, **R_0_**, defined as the average number of descendants an individual parasite would have at the beginning of bloodstream infection in the absence of any host response to the parasite. As long as *T_D_*_μ_ (duration of the merozoite stage)≪the duration of the infected RBC stage, then by probability arguments independent of model details [41],
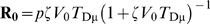
(1)


Here *V*_0_ is the initial density of the RBC population vulnerable to the parasite species, *ζ* is the binding affinity of merozoites to RBCs, and *p* is the average number of merozoites released per bursting schizont. (For *P. falciparum*, *V*_0_ = normal basal RBC count = 5×10^6^ µl^−1^; for *P. vivax*, *V*_0_ = the basal reticulocyte count = 6.25×10^4^ µl^−1^.) Experimental evidence suggests that *T_D_*_μ_ is just minutes; we take it as 0.1 h [42]. Data from the time series of parasitemia in neurosyphilis patients infected with *P. vivax* for malariatherapy [43] suggest that for *P. vivax*, **R_0_**∼15. Similar data for *P. falciparum* from neurosyphilis [44] and other patients [45], as well as direct photographic evidence from studies of bursting schizonts [46] suggest **R_0_** and p are both ≥15 for that species as well. Knowing **R_0_**, *T*_Dμ_, *p*, and *V*_0_ determines *ζ*. Because we wanted to simulate infections with parasite growth rates seen clinically, we used *p* = 16 with **R_0_** = 15 for both species. The characteristic invasion time of an RBC by a merozoite in this model, (ξ*V*_0_)^−1^, is 24 seconds. Equation 1 is consistent with our CODEs in that if the immune response is absent, a steady state with a nonzero parasite count exists only if **R_0_**>1 [41].

We simulated infections for up to one year after primary release of merozoites (when they enter the blood from the liver), unless the parasite population was cleared by the host first, or unless the RBC count of the host declined to less than 60% of the normal basal count of 5×10^6^ µl^−1^ (in which case it is assumed that the host died). For each type of parasite and immune response, we considered four model RBC source dynamics, described in detail in the Model section: (1) RBC production *increases* to match the RBC loss due to infection (compensatory response), (2) the RBC production rate strongly *declines* in response to RBC loss, down to 65% of the basal rate (severe dyserythropoiesis), (3) a more moderate dyserythropoiesis occurs, with RBC production rate reduced down to 75% of the basal rate, and (4) RBC production remains fixed at the basal rate. We cannot emulate the full complexity of erythopoietic response in malaria, but these choices represent plausible modalities in that response. Altogether, for each of the two species of parasites we conducted about 3.8×10^4^ simulations with varying values of the erythropoietic and immune parameters and targets for an antibody-naïve host. In addition, for model *P. vivax* infections, we considered about 8×10^3^ simulations in which a pre-existing level of antibody from a previous infection was present; details are in the Model section.

**Figure 2:**
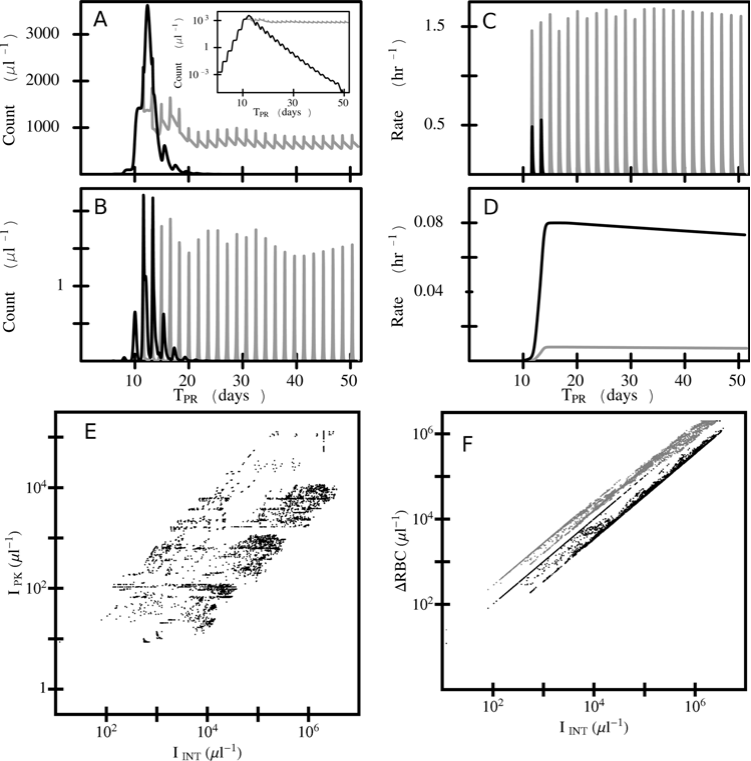
Some typical simulation results. Panels (A)–(D) show time series for several quantities for two model *P. vivax* infections with SD*_I_* = 2.4 h. *T*_PR_ is the time since primary release. For both, the source rate of RBC production is fixed, the innate response targets the last half of IBC development (late trophozoite-schizont stage), the host is antibody naïve, and the antibody response targets any part of the IBC stage. Black curve: *A*_th_ = 50 µl^−1^, *ξ* = 0.2 µl h^−1^ for the innate response, and *A*_th_ = 0.5 µl^−1^, *ξ* = 0.008 µl h^−1^, 1/*λ*_Att_ = 8,760 h for the antibody response. Gray curve: *A*_th_ = 50 µl^−1^, *ξ* = 1.0 µl h^−1^ for the innate response, and *A*_th_ = 0.5 µl^−1^, *ξ* = 0.0008 µl h^−1^, 1/*λ*_Att_ = 8,760 h for the antibody response. Where black and gray overlap, black is shown. (A) IBC count (inset shows the time series on a semilog plot), (B) merozoite count, (C) clearance rate of IBCs by the innate response, (D) clearance rate of IBCs by the antibody response, (E) the peak IBC count (*I*_PK_) plotted against the integrated IBC count (*I*_INT_) for all of the *P. vivax* simulations with fixed RBC production rate, (F) the RBC deficit (ΔRBC) plotted against the integrated IBC count (*I*_INT_) for all of the *P. vivax* simulations with fixed RBC production rate (black) and all of the *P. vivax* simulations with moderate dyserythropoiesis (gray).

Figure 2 shows results from typical simulations with an antibody-naïve host. The parasitemia and immune factor time series shown in panels A–D compare 2 model *P. vivax* infections over the first 52 days after the primary release of parasites from the liver into the bloodstream. For both simulations, the model parameters for the parasites are the same, the rate of RBC production is fixed, and the immune responses have the same targets, but the binding affinities to those targets differ between the 2 models. In Figure 2A, note that the lower peak count of IBCs (infected RBCs) occurs in the model with the larger, more sustained innate-response-driven clearance rate, but it is in the other model, with the larger antibody-driven clearance rate, that the infection is completely cleared during this time span. (Note: clearance rate = binding affinity to target×nominal level of the component that attacks the target; see Model section.) Figure 2E plots peak IBC count (*I*_PK_) against integrated IBC count (*I*_INT_, the total number of IBCs produced during the infection) for all of the *P. vivax* models with a fixed rate of RBC production. *I*_PK_ versus *I*_INT_ plots for the other parasite species and erythropoietic responses are similar (see Figure S1 for further examples). To indicate severity of anemia, we use the RBC deficit, ΔRBC, defined as 5×10^6^ µl^−1^ – (minimum RBC count during infection). Figure 2F plots ΔRBC against I_INT_ for all of the *P. vivax* simulations for an antibody-naïve host with a fixed rate of RBC production (black points) and moderate dyserythropoeisis (gray points). Note that for the same *I*_INT_, ΔRBC tends to be (but is not always) one-half to one order of magnitude higher (i.e., anemia is much more severe) for the models with moderate dyserythropoeisis. Results for *P. falciparum* infections and for *P. vivax* infections with pre-existing antibodies are similar (see Figure S1).

### Effects of Variations in Model Parameters on Simulation Outcomes

**Figure 3:**
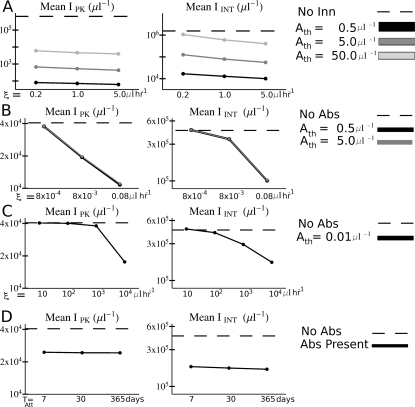
Overall variations in parasitemia with changes in the model immune parameters. The host was antibody-naïve in all simulations. (A) Plot of the peak (*I*_PK_) and integrated (*I*_INT_) parasitemia averaged over all simulations (results for *P. vivax* and *P. falciparum* combined) with the corresponding values of target binding affinity *ξ* and antigen detection threshold *A*_th_ for the innate responses. The dotted lines show the averages over simulations with no innate response. (B) Plot of *I*_PK_ and *I*_INT_ averaged over all simulations with the corresponding values of target binding affinity *ξ* and threshold trigger sensitivity *A*_th_ for the antibody responses that do not target merozoites. (C) Plot of *I*_PK_ and integrated *I_INT_* averaged over all simulations with the corresponding values of target binding affinity *ξ* for the antibody responses that target merozoites. (D) Plot of *I*_PK_ and integrated *I*_INT_ averaged over all simulations with the corresponding values of attacker decay time *T*_Att_ for all antibody responses. In (B), (C), and (D), the dotted lines show the averages over all simulations with no antibody response. Note: In all panels, the vertical scale is logarithmic.

Peak and integrated parasitemia in individual simulations depend on details of the interactions within the CODE model, but the plots in Figure 3 indicate the trends in simulation outcomes with changes in the immune response parameters in antibody-naïve hosts. Note in panel A that an order of magnitude change in the trigger level for the innate response leads to an order of magnitude change in *I*_PK_ and *I*_INT_ on average. *I*_PK_ and especially *I*_INT_ are sensitive to changes in the binding affinity of the antibody response to its target (panels B and C), on average, but less so to the decay time of the attacker component of the antibody response (panel D). Note, too, in comparing panels B and C, that in order to achieve the same degree of control of parasitemia, the binding affinity of an antibody response targeting only merozoites must be many orders of magnitude greater than one targeting an IBC stage. Because the maximum nominal concentration of the attacker component is fixed at 10 µl^−1^ for all antibody models (see Model section), the corresponding clearance rates must also differ by many orders of magnitudes for the same degree of parasitemia control. This might be expected since free merozoites either invade an RBC or else are cleared within a few minutes, and thus are exposed to antibodies for a much shorter time than are IBCs. Though the model antibody response against merozoites works by removing them from the system, the model is also plausible for antibodies which block merozoites from infecting RBCs, since, as discussed above, merozoites are so quickly removed from the blood.

### Antibodies But No Innate Response

In this section we consider simulated infections in which the host has no innate immune response; in the next sections we show that, as a class, model infections in which the host has an innate response have qualitatively different behavior. This contrast might be expected based on Figure 3A, which shows that the peak parasitemia *I*_PK_ for simulations without an innate response (but with an antibody response) is orders of magnitude higher, on average, than for simulations with an innate response even for the smallest innate-parasite binding affinity and largest threshold of innate activation considered. An examination of infection outcomes without an innate response distinguishes outcomes to which it is likely to be most and least crucial, and is not an academic exercise: similar outcomes might occur if the parasite were able to evade or suppress innate response such that it became ineffective in eliminating parasites.

**Figure 4:**
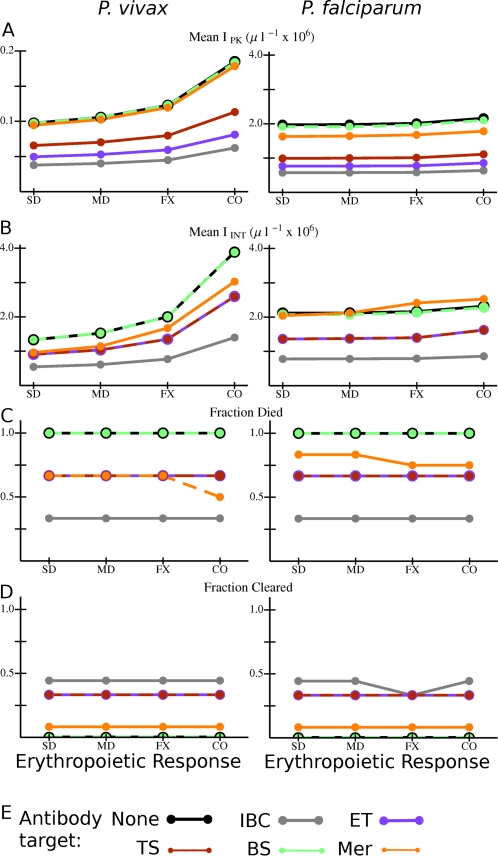
Overall variation in outcome with different combinations of *Plasmodium* species, erythropoietic and antibody responses for model infections in antibody naïve hosts with no innate response. (A) *I*_PK_ and (B) *I*_INT_ averaged over all simulations with the given combination of species, erythropoietic response, and antibody target. (C) Fraction of all simulations in which the host died, and (D) fraction of simulations in which the host cleared the parasite for the given combination of species, erythropoietic response, and antibody target. (E) Color code for the data points and lines. Abbreviations for antibody responses: None: no antibodies present; IBC: any IBC stage; ET: early trophozoite; TS: late trophozoite-schizont stage; BS: bursting schizont; Mer: merozoite. Abbreviations for erythropoietic responses: SD: severe dyserythropoiesis; MD: moderate dyserythropoiesis; FX: RBC production rate fixed at basal rate; CO: compensatory erythropoiesis. Lines are just to guide the eye. If the data points and connecting lines for two or more antibody responses overlap in the plot, the lines are dashed to reveal all the responses present. Note in Panel A that the vertical scale on the plots is different between the two species.

Figure 4 shows the values of several quantities averaged over all simulated infections for each type of model antibody and erythropoietic response that we considered. The host lacks an innate response, and is antibody naïve. As noted above, results for antibody responses which attack the merozoite stage cannot be directly compared with those which attack IBC stages since antibodies against merozoites must induce a much higher clearance rate to be similarly effective. However, note that for all types of antibody responses, the peak and integrated parasitemia tend to be higher with compensatory erythropoiesis than with dyserythropoiesis or with RBC production fixed at the basal rate, strikingly so for *P. vivax* infections. Peak parasitemia *I*_PK_ in model *P. falciparum* infections is ∼10×*I*_PK_ in *P. vivax* infections (Figure 4A), as would be expected since a much smaller subset of the RBC population can be infected by the latter species. However, Figure 4B shows that I_INT_ in *P. vivax* infections is of the same order of magnitude as in *P. falciparum* infections. (The same is also true for the ΔRBC; see Figure S2A.) Furthermore, for a given combination of antibody and erythropoietic responses, the fraction of simulated infections which ends in host death by catastrophic anemia is nearly the same for the two species (Figure 4C), except that the fraction of infections with host antibodies to merozoites ending in death is lower for *P. vivax* than for *P. falciparum*. (Incidentally, for infections with antibodies to merozoites of either species, this fraction is lower with compensatory erythropoiesis than dyserthropoiesis.) But Figure 4A and 4B show a major difference between the infection dynamics produced by the two species: the ratio *I*_PK_/*I*_INT_ in model *P. falciparum* infections is just under 1, while in *P. vivax* infections it is closer to 1/20. This is the case because, if host death occurs, it occurs within 2–5 weeks after primary merozoite release in *P. falciparum* infection, but requires 8–15 weeks in *P. vivax* infection (see Figure S3). Thus, much of the integrated parasitemia in *P. falciparum* infection in this set of models is due to a final burst of parasite production before host death. Another pattern is apparent in Figure 4 among the antibody responses which target an IBC stage: the longer the duration of vulnerability, the better the response is at controlling parasitemia, reducing risk of host death, and inducing parasite clearance in infections with either species. For example, note in Figure 4D that 100% of infections with an antibody response that attacked only the bursting schizont stage (with no innate response present) ended in host death, but only about 30% of those with antibodies that attacked infected RBCs of all stages died. Furthermore, the latter, broad antibody response resolved nearly 45% of infections by clearance of the parasite within one year (Figure 4D). For model infections in which parasites are cleared, clearance tends to occur within 2–6 weeks after primary release; see Figure S3.

**Figure 5:**
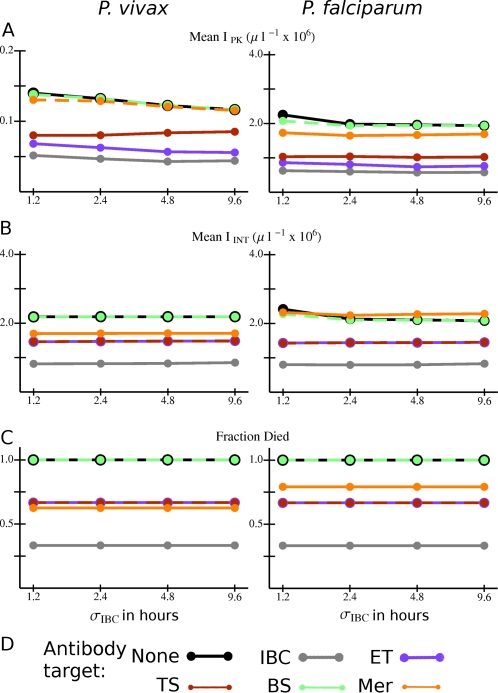
Overall variation in outcomes with different combinations of *Plasmodium* species, antibody responses, and standard deviations in the intraerythrocytic development time (*σ*_IBC_) for model infections in antibody naïve hosts with no innate response. (A) *I*_PK_ and (B) *I*_INT_ averaged over all simulations with the given combination of species, *σ*_IBC_, and antibody target. (C) Fraction of all simulations in which the host died for the given combination of species, *σ*_IBC_, and antibody target. (D) Color code for the data points and lines. Abbreviations for antibody responses as in Figure 4. Lines are just to guide the eye. If the data points and connecting lines for two or more antibody responses overlap in the plot, the lines are dashed to reveal all the responses present.

In the absence of an innate response, infection outcomes were rather insensitive to differences in the standard deviation of the development time of the parasite within the IBC (*σ*_IBC_) (see Figure 5 and Figure S2B). However, a shorter σ_IBC_ was associated with a slightly higher peak and integrated parasitemia in model *P. falciparum* infections (Figure 5A and 5B). Peak parasitemia in *P. vivax* infection tended to be higher for a shorter *σ*_IBC_ as well, except with antibodies that target the late trophozoite-schizont stage (Figure 5A).

As mentioned above, we also did simulations that assumed a level of pre-existing antibodies in the host. For this set of simulations, we assumed that the species was *P. vivax*, and that the antibodies could attack any IBC stage. (For more details see Model section below.) We found that if there was no innate response, the results were very similar to those shown in Figure 4 for this type of antibody response; see Figure S4.

### Simulations with an Innate Response Present

**Figure 6:**
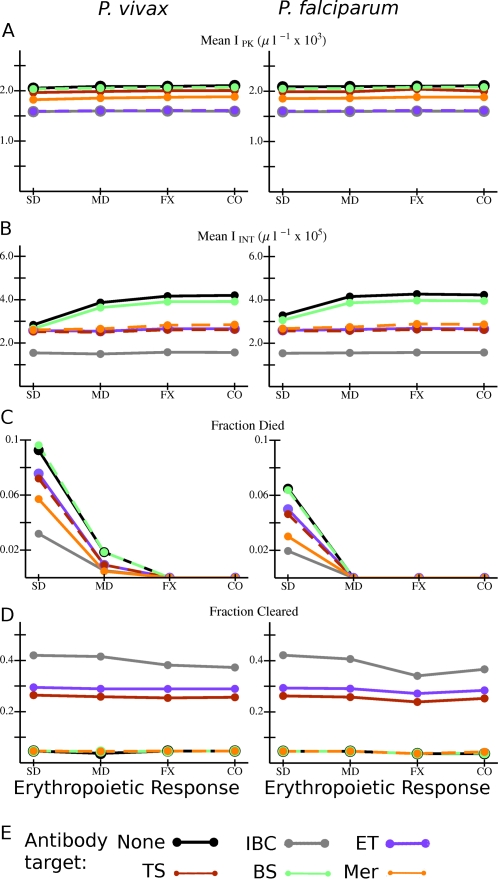
Overall variation in outcome with different combinations of *Plasmodium* species, erythropoietic and antibody responses for model infections in antibody naïve hosts that mount an innate response. (A) *I*_PK_ and (B) *I*_INT_ averaged over all simulations with the given combination of species, erythropoietic response, and antibody target. (C) Fraction of all simulations in which the host died, and (D) fraction of simulations in which the host cleared the parasite for the given combination of species, erythropoietic response, and antibody target. (E) Color code for the data points and lines. Abbreviations as in Figure 4. Lines are just to guide the eye. If the data points and connecting lines for two or more antibody responses overlap in the plot, the lines are dashed to reveal all the responses present. (Note: the vertical scale for panel D is different from one in Figure 4D.)

Figure 6 is the same as Figure 4, in that it shows the values of several quantities averaged over all simulated infections for each type of model antibody and erythropoietic response, except that the host also has an innate response. (As noted above, the model innate responses can attack either the early trophozoite stage, the late trophozoite/schizont stage, or the entire intraerythrocytic stage.) Hosts are antibody naïve. Many differences between the two figures are apparent. (Note the one order of magnitude difference in the vertical scales between Figures 4 and 6 in panels A and B.) First, parasitemia, on average, is much better controlled by the host if an innate response is present: *I*_PK_ (panel A) is reduced a factor of 100–1000 and *I*_INT_ (panel B) by a factor 5–10. The presence of an innate response generally diminishes the effects of erythropoietic response on I_PK_ and *I*_INT_, notably of compensatory response on *P. vivax*. The striking exception is that here severe diseryrthropoiesis with no antibody response, or an antibody response only against bursting schizonts, decreases *I*_INT_ for both species. On the other hand, the fraction of hosts surviving infections is sensitive to the nature of the erythropoietic response: only with dyserythropoiesis did infections with model innate responses end in host death (panel C). (This sensitivity to erythropoietic response is mirrored in ΔRBC; see Figure S5A.) Dyserythropoiesis with a broad antibody response slightly increased the fraction of infections cleared (Figure 6D), but, even with severe dyserythropoiesis, for all model antibody types, the fraction of infections ending in host death was much lower if the host mounted an innate as well as an antibody response. As with Figure 4, for antibody responses that target an IBC stage, the longer the duration of the vulnerable stage, the lower the peak and integrated parasitemia, the lower the fraction of infections ending in host death, and the higher the fraction of infections in which the parasite is cleared.

**Figure 7:**
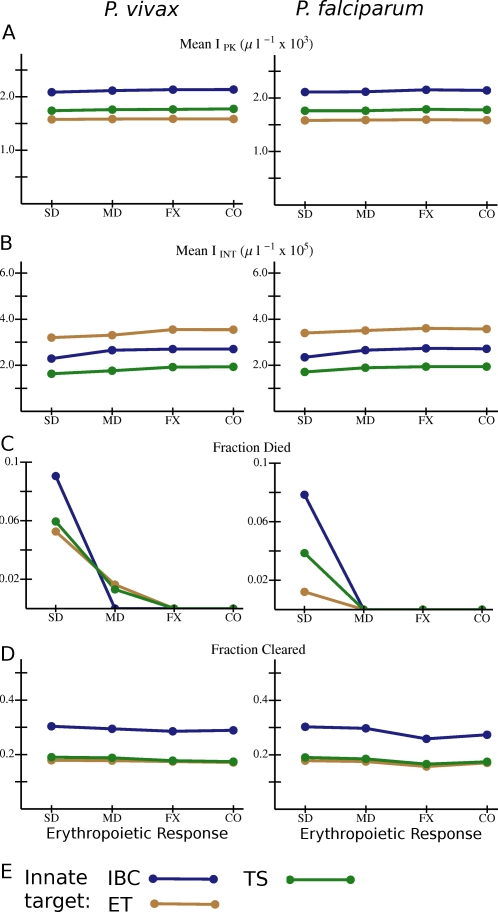
Overall variation in outcome with different combinations of *Plasmodium* species, erythropoietic and innate responses for model infections in antibody naïve hosts that mount an innate response. (A) *I*_PK_ and (B) *I*_INT_ averaged over all simulations with the given combination of species, erythropoietic response, and innate immunity target. (C) Fraction of all simulations in which the host died, and (D) fraction of simulations in which the host cleared the parasite for the given combination of species, erythropoietic response, and innate target. (E) Color code for the data points and lines. Abbreviations for innate responses: IBC: any IBC stage; ET: early trophozoite; TS: late trophozoite-schizont stage. Abbreviations for erythropoietic responses as in Figure 4. Lines are just to guide the eye. If the data points and connecting lines for two or more antibody responses overlap in the plot, the lines are dashed to reveal all the responses present.

Figure 7 again shows values of several quantities averaged over all simulated infections (in antibody naïve hosts with innate responses) for each type of innate response and erythropoietic response. (The averaging process includes averaging over all model antibody responses for the given innate and erythropoietic response.) For all three innate responses studied, the risk of host death is highly sensitive to the nature of the erythropoietic response. (So is ΔRBC; see Figure S5B.) If the early trophozoite IBC stage is targeted by the innate response, *I*_PK_ tends to be lower than for the other two types of innate targets (Figure 7A). However, *I*_INT_ is lower if the late trophozoite-schizont stage is targeted (Figure 7B). The early trophozoite is a slightly better target than the late trophozoite-schizont in terms of reducing the risk of host death (Figure 7C) but slightly worse in terms of enhancing clearance (Figure 7D). The broad response, covering both IBC stages, is best at enhancing clearance but, with severe dyserythropoiesis, worst at reducing the risk of host death. Because the late trophozoites and schizonts are believed to be the parasite stages most vulnerable to fever [47], we will return to these points in the Discussion.

Another striking aspect of both Figures 6 and 7 is that infections with *P. vivax* and *P. falciparum* have very similar outcomes, on average, when the host can mount an innate attack on the parasite. We found that the differences between the species in the values of *I*_PK_ and *I*_INT_ averaged over a given immune and erythropoietic response were sometimes as small as 1%. One major difference between the species is that, with a dyserythropoeitic response, the fraction of infections ending in host death is higher for *P. vivax* infections than for *P. falciparum* infections (Figures 6C and 7C). This result holds true, on average, for each type of antibody response and innate response. Apparently, since *P. vivax* attacks the youngest RBCs, even a low-level parasitemia can deplete the number of RBCs that mature, which in turn can more often or more quickly lead to catastrophic anemia if the rate of RBC production is reduced. On the other hand, in *P. falciparum* infections, low-level depletion of the general RBC pool by the parasite may be less critical. (Even for infections with compensatory erythropoiesis or a fixed rate of RBC production, in which the outcome tends to be much less severe for the host, ΔRBC is higher for hosts infected with *P. vivax* than with *P. falciparum*, for all types of immune responses; see Figure S5). With a host innate response, with either parasite species and each type of antibody and erythropoietic response, host deaths that ended infections occurred at least 20 weeks after primary merozoite release, on average, much longer than if no innate response were present; see Figure S6. With infections resolved by parasite clearance, for each antibody and erythropoietic response the average time from primary release to clearance was 1–6 weeks; see Figure S6).

Among the simulations of *P. vivax* infections in hosts with pre-existing antibodies to IBCs of all stages, we found essentially the same trends as in Figure 7: for all model erythropoietic responses, the innate response which attacks the late trophozoites and schizonts is more effective, on average, than the other two in reducing *I*_INT_ and ΔRBC, while dyserthyropoiesis greatly aggravates ΔRBC and increases the frequency of host death, even if it also slightly increases the frequency of parasite clearance (see Figure S7).

### Consistency of Development Time and Infection Outcome with an Innate Response

**Figure 8:**
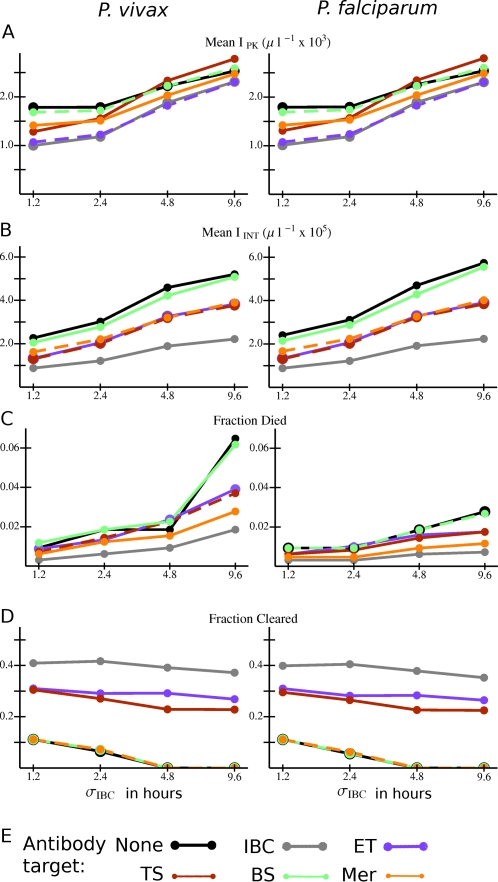
Overall variation in outcome with different combinations of *Plasmodium* species, standard deviations in the intraerythrocytic development time (*σ*_IBC_), and antibody responses for model infections in antibody naïve hosts that mount an innate response. (A) *I*_PK_ and (B) *I*_INT_ averaged over all simulations with the given combination of species, *σ*_IBC_, and antibody target. (C) Fraction of all simulations in which the host died, and (D) fraction of simulations in which the host cleared the parasite for the given combination of species, *σ*_IBC_, and antibody target. (E) Color code for the data points and lines. Abbreviations for antibody responses as in Figure 4. Lines are just to guide the eye. If the data points and connecting lines for two or more antibody responses overlap in the plot, the lines are dashed to reveal all the responses present.

**Figure 9:**
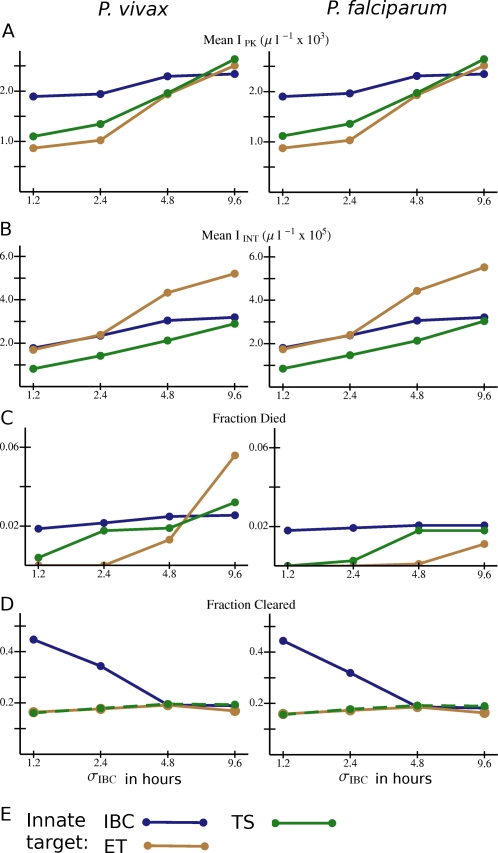
Overall variation in outcome with different combinations of *Plasmodium* species, standard deviations in the intraerythrocytic development time (*σ*_IBC_) and innate responses for model infections in antibody naïve hosts that mount an innate response. (A) *I*_PK_ and (B) I_INT_ averaged over all simulations with the given combination of species, *σ*_IBC_, and innate immunity target. (C) Fraction of all simulations in which the host died, and (D) fraction of simulations in which the host cleared the parasite for the given combination of species, σ_IBC_, and innate immunity target. (E) Color code for the data points and lines. Abbreviations for innate responses as in Figure 7. Lines are just to guide the eye. If the data points and connecting lines for two or more antibody responses overlap in the plot, the lines are dashed to reveal all the responses present.

As shown in Figure 5, if no host innate response is present, parasitemia is either insensitive to *σ*_IBC_ or higher for smaller *σ*_IBC_, depending on the parasite species and antibody response. The situation is strikingly different if an innate response is present. Figure 8 shows the values of several quantities from simulations with an innate response averaged over the type of antibody response and *σ*_IBC_, and Figure 9 shows the values of the same quantities averaged over the type of innate response and *σ*_IBC_. (In both figures, results are for antibody naïve hosts.) Note in these two figures that *I*_PK_ and *I*_INT_ decline with decreasing σ_IBC_, on average, for each type of antibody response, for each type of innate response, and for both parasite species. (The same is true for the RBC deficit ΔRBC; see Figure S8.) Apparently, the faster rate of schizont bursting which occurs with smaller *σ*_IBC_ triggers the innate response more quickly and keeps parasitemia lower, on average. The fraction of infections ending in host death also declines with decreasing *σ*_IBC_, though not necessarily monotonically (Figures 8C and 9C). (For simulations that end in host death, the time from primary merozoite release to host death tends to increase with smaller *σ*_IBC_, on average, though this effect is not monotonic; see Figure S9.) The antibody response that attacks all IBC stages and the innate response that attacks late trophozoites and schizonts have, on average, lower *I*_INT_ and ΔRBC than the other model antibody and innate responses, respectively, for all values of *σ*_IBC_ (Figures 8B and 9B, and Figure S8). The fraction of infections ending in parasite clearance has a somewhat complicated dependence on *σ*_IBC_. If the innate response can attack the entire IBC stage, then this fraction increases with decreasing *σ*_IBC_, but is insensitive to the value of *σ*_IBC_ for the other two model innate responses (Figure 9D). Interestingly, for some types of model antibody response, simulations with an innate response never lead to parasite clearance unless *σ*_IBC_<4.8 hours (Figure 8D).

For the set of simulations done for *P. vivax* infections assuming a level of pre-existing antibodies to the entire IBC stage, the variations of *I*_PK_, *I*_INT_, ΔRBC, fraction of infections ending in host death, and fraction of infections ending in clearance of parasite with σ_IBC_ were similar to the trends observed in the antibody naïve hosts (see Figure S10).

## Discussion

Our results provide some insights into real malaria infections and parasite biology, despite the vast simplifications involved in modeling immune and erythropoietic responses. First, anemia did not lead to death in any simulation unless there was either dyserythropoiesis or an absence of innate response. Apparently, for *P. vivax* or *P. falciparum* infection to produce catastrophic anemia in the presence of a fast-activating innate response, one of the following must happen: (a) dyserythropoiesis, (b) a weakening of the innate response, coupled with an adaptive response which fails to check parasite growth, or (c) sustained destruction and/or sequestering of uninfected RBCs (“bystander effect”). We did not consider a bystander effect directly, but note that dyserythropoiesis could amplify the resulting anemia. We cannot say anything directly about host death due to other causes, but we note that infections with high parasitemia (*I*_PK_>10^4^ µl^−1^ or *I*_INT_>10^6^ µl^−1^) were the ones with no or very weak innate response (as illustrated in Figures 3 and 4). Although control of the magnitude of peak parasitemia was dominated by the presence or absence of an innate response, our results suggest that an antibody response which targets a significant duration of the intraerythrocyte stage will reduce anemia and integrated parasitemia and boost the likelihood that a host will clear the infection. Even an antibody response that targets only merozoites would reduce parasitemia if the reciprocal of the clearance rate is greater than the characteristic time for a merozoite to find and invade an RBC (as suggested by Figure 3D).

It is hardly surprising to find that the antibody response with the widest possible range of targets produces the lowest frequency of host death and the highest frequency of parasite clearance (Figures 4, 6, and 8). Our results emphasize the different durations over which stage-specific antigens are exposed to antibody attack—an antibody response that targets merozoites or bursting schizonts is much less effective, for instance—and the value of an array of antibody responses to an array of stage-specific antigens. With an immune response that includes innate as well as antibody components, rather than antibody alone, the frequency of host death is lower (compare Figures 6C and 4C), and, as with real malaria infections, more infections persist for long periods at low parasitemia.

It is the gametocytes that allow parasite genomes to be transported between hosts, as modified by meiotic recombination in the mosquito, and the conditions that regulate gametocyte production and infectivity remain unknown. However, if the production or infectivity of gametocytes is proportional to either peak or integrated parasitemia, it appears unlikely that inducing dyserythropoiesis would provide a transmission advantage to either *P. vivax* or *P. falciparum*. Our calculations suggest that a dyserythropoietic response to infection, compared to compensatory erythropoiesis or RBC production held at the basal rate, would suppress I_PK_ and I_INT_, most dramatically in hosts lacking an innate response to *P. vivax* (Figure 4), but also for I_INT_ for both species, in hosts with both immune-response components engaged (Figure 6). In the latter case, though dyserthropoiesis can slightly increase the frequency of parasite clearance, it can substantially increase the risk of severe anemia and host death. Our model does not consider the possibility that induction of dyserythropoiesis might be accompanied by reduced effectiveness of immune responses, however.

It is possible that enhanced parasitemia enhances propagation of the parasite's genome, presumably by boosting gametocytemia, but it is also possible that enhanced parasitemia or gametocytemia triggers an immune response that limits transmission, and thus that the *regulation* of parasitemia or gametocytemia could benefit the parasite in an evolutionary sense. In vitro experiments have shown that typical febrile temperatures kill intraerythrocytic parasites in the last but not the first 24 hours of their development [47]. Interestingly, in our CODE models, I_INT_ (and thus the total number of merozoites produced) is lower on average when the innate response attacks the late trophozoites and schizonts rather than the early trophozoites, a result that is a consequence of the model *dynamics*. If there are parasite adaptations that involve direct or indirect regulation of parasitemia that enhance gametocyte transmission, then the parasite may actually benefit by the vulnerability of the second half of the IBC stage to fever.

When an innate response is present in the model, compensatory erythropoietic response leads to nearly the same *I*_PK_ and *I*_INT_, on average, as RBC production fixed at the basal rate. Our results show slightly lower anemia with a compensatory response than with a fixed RBC production rate, in the presence of innate immunity (Figure S5), but differences between the two in any outcome are remarkably few and small, which implies that, in the presence of an innate immune response, a compensatory erythropoietic response confers little or no advantage or disadvantage to either host or parasite. This marks a contrast to some of our results with an earlier model [41] in which, specifically to examine constraints on immune responses, we included only parasite and RBC dynamics. In that context, compensatory response boosted parasitemia and accelerated the onset of catastrophic anemia in a set of model *P. vivax* infections. Our conclusion here might change if there were long-term evasion or suppression of the innate response by the parasite—since, in the absence of an innate response, compensatory response boosts parasitemia (Figure 4)—or if removal of uninfected RBCs boosted RBC production well above rates that would compensate for infection-induced RBC loss [32]. Overall, our results on the role of erythropoietic response point to larger questions of how trade-offs of compensatory and dyserythropoietic responses might balance, and how differences between individuals translate into differences in populations; the evolutionary aspects involve dynamics on multiple scales, to be incorporated in future work [48].

Our results showed lower *I*_PK_ and *I*_INT_ for smaller values of the standard deviation in the IBC development time, *σ*_IBC_, provided an innate response was present (see Figures 8 and 9). This was true for all innate and antibody targets and for both species, suggesting that a very tight synchronization of merozoite release by bursting schizonts would not benefit the parasite if parasitemia correlates with transmission probability. Furthermore, tight synchronization benefits the host overall, through enhanced clearing of infections, and reduced RBC deficits and frequencies of death. It has been suggested, however, that the release of prostaglandins or other factors by bursting schizonts during synchronization may suppress immunity [20], an effect we did not consider. Furthermore, we assumed that the model immune responses were not overwhelmed by large increases in parasitemia, which should be true if the response is provided by fever directly, or by a large reservoir of antibody-producing cells. However, a limited pool of cells that target and kill parasites could perhaps be overwhelmed by a synchronized burst. And we did find that if the innate response is absent, parasite synchronization yields little benefit for the host. Again, these results indicate trade-offs that merit further investigation [14].

Finally, we address the relative abilities of *P. vivax* and *P. falciparum* to cause catastrophic illness. In our previous report—on a model which incorporated erythropoeitic but not immune responses—we inferred that without an effective immune response, *P. vivax* would be highly lethal, even for values of **R_0_** just above 1, due to its depletion of reticulocytes and the consequent eventual lack of replacements for senescent RBCs [41]. Among the results of our new simulations, done with active immune responses, two trends stood out when comparing the outcome of simulated *P. vivax* infections with those caused by *P. falciparum*: (1) for the same host immune and erythropoietic responses, *P. vivax* produces ΔRBC values equal to or even greater than those of *P. falciparum*, and (2) under similar conditions of either dyserythropoiesis or an ineffective innate response, the fraction of simulations ending in catastrophic anemia was just as high or higher for *P. vivax* as for *P. falciparum*. In fact, as can be seen in Figures 6C and 7C, no *P. falciparum* infections with both innate response and moderate dyserythropoiesis ended in host death, while a small fraction of *P. vivax* infections with those characteristics did. Though real *P. vivax* infections are rarely fatal, they are debilitating and trigger extremely strong immune responses (such as the paroxysm response described above). Our new results suggest that if these responses truly clear the parasite from the blood, their strength is not inappropriately aggressive, and may be a result of strong selective pressures from *P. vivax* malaria on human innate and adaptive immune systems. Indeed, recent reports of *P. vivax* malaria with severe organ complications [49], splenic infarction [50], and retinal hemorrhage [51] suggest that *P. vivax* can induce “malignant” illness in some individuals. There is also an increased risk of low birthweight in infants born to mothers with *P. vivax* malaria [52].

In both evolutionary and proximate terms, immune responses shaped by *P. vivax* infections are likely to be partly but not completely appropriate for *P. falciparum* infections, and vice versa [53],[54]. It may be that *P. falciparum* tends to cause more severe illness often than *P. vivax* because it is more effective at evading, suppressing or otherwise subverting the human immune responses on a population average. These is also the possibility that dyserythropoiesis is worse in *P. falciparum* infection. How these and other factors play off in determining the relative transmission success of the two species over multiple time scales is an intriguing topic for future investigation.

## Model

### General Considerations

Our models are composed of compartmentalized ordinary differential equations (CODEs) and are closely similar to models in our previous work [41],[54] but include immune responses. CODEs are designed to simulate an ensemble of cells that, on average, take a certain duration *T*_D_ to pass through a development stage. The standard deviation in development time is
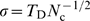
(2)where *N*_c_ is the number of compartments used to model the development stage [55],[56]. We stress that *T*_D_ and *σ* are the tangible quantities.

Figure 1 is a schematic representation of the model. RBCs emerge from the bone marrow, and, if uninfected, circulate for 120 days [57]. We assume that RBCs of all age cohorts are vulnerable to *P. falciparum*, but that *P. vivax* attacks only reticulocytes. The rate of RBC loss due to infection can feed back to the RBC source, to increase (compensatory response) or decrease (dyserythropoietic response) the RBC production rate. The immune components of our model are based on simple ideas from immunology, and resemble models of immune response in both viral infections [58]–[60] and malaria [34]. An antigen signal over a threshold level triggers an initially self-amplifying (and eventually self-limiting) *actuator* phase, which in turns initiates either an *attacker* phase, which directly removes parasites, or a *growth* phase, which models the division of B or T cells, in which case the product of the growth phase initiates the attacker phase. The two-stage actuator-attacker response emulates a quick-acting but short-lived innate response, while the three-stage actuator-growth-attacker response emulates a slower-acting adaptive response, such as the production of antibodies against a target. As real antibody responses have control mechanisms that keep them from reaching destructive levels (reviewed in [61]), in our three-stage model immune response the attacker phase is also self-limiting.

We now introduce the equations of motion for the system, starting with those for the parasite population. Then we consider those for the RBC population and the immune factors.

### Parasite Population Dynamics

Let *T*_DI_ be the average duration of the infected RBC stage, with standard deviation *σ*_IBC_. Then *N*_cI_ = *T*_DI_^2^/*σ*_IBC_. is the number of compartments needed to described the IBC development. Let *I_n_* be number of infected RBCs per µl in compartment *n*. Then
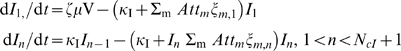
(3)


Here κ_I_ = *N*_cI_/*T*_DI_, *μ* is the merozoite density, *ζ* is the binding affinity between the merozoites and vulnerable RBCs, and *V* is the total density of vulnerable RBCs for a given species: for *P. vivax*, *V* = uninfected reticulocyte count, and for *P. falciparum*, *V* = *E*_T_, the total uninfected RBC count. The infected RBCs (IBCs) could themselves be vulnerable to several immune system components: *Att_m_* refers to the density of the attacker phases of component *m*, and *ξ_m_*_,*n*_ is the binding affinity between the attacker phase of immune component m and the parasite in compartment *n*. We allow *ξ* to have dependence on *n* in order to emulate an age-specific immune attack on IBCs. This is valid within the CODEs formalism provided one realizes that if *N*_sub_ is the number of compartments associated with a subinterval of the IBC stage, the standard deviation in the time any one IBC spends in the subinterval is ∼*T*_DI_ *N*_sub_^1/2^/*N*_cI_. We took *T*_DI_ = 48 h for both *P. vivax* and *P. falciparum* [1]. For each immune and erythropoietic response, we evaluated the model's behavior for several values of *σ*_IBC_ (given in Table 1). The discussion after Equation 1 gives the details on how the value of *ζ* is set.

**Table 1:** Values of Parameters for Model Parasites.

Species or Strain	*p*	R_0_	Standard deviation *σ*_IBC_ in IBC duration (h)
*P vivax*	16	15	1.2, 2.4, 4.8, or 9.6
*P falciparum*	16	15	1.2, 2.4, 4.8, or 9.6

We use just one compartment for the merozoite stage, due to its short survival time in the blood, *T*_Dμ_ = *σ*_μ_ = 0.1 h [42]:
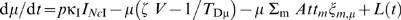
(4)


Here, *p* is the number of merozoites released per bursting IBC after development and asexual division are completed within an IBC; the choices for *p* are discussed below. Parameter *ξ_m_*_,*μ*_ is the binding affinity of immune response *Att_m_* to merozoites. *L*(*t*) is the infusion of primary merozoites of the given species from the liver into blood, which is believed to happen quickly and to involve 10^4^–10^5^ merozoites. For our simulations, the initial time (*t* = 0) corresponds to the release of the first parasite from the liver. For simplicity, we took *L*(*t*) = 0.002 (µl h)^−1^, for the first hour, (corresponding to a release of 10^4^ primary merozoites into a blood volume of 5×10^6^ µl) and then zero afterwards. We redid some simulations with 10^5^ primary merozoites released, but this had little effect on the outcome (results not shown).

In *P. falciparum* malaria, the IBCs sequester onto blood vessel walls during the second phase (last 24 hours) of parasite intra-RBC development. Sequestered parasites can readily release large numbers of merozoites that attack neighboring RBCs, however [46]. Thus, in our model, we can consider the IBC compartments to include sequestered as well as freely-circulating IBCs.

### RBC Population Dynamics

The RBC development chain is divided into three parts. We first consider RBCs attacked by *P. falciparum*; the CODEs for reticulocytes are
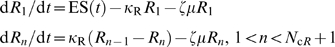
(5)For mature RBCs:
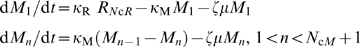
(6)For senescent RBCs:
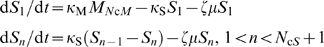
(7)(We separate the senescent stage from the mature stage to allow for possible modeling of *P. malariae* infections, in which mainly senescent RBCs are attacked.) Here, *κ*_R_ = *N*_c*R*_/*T*_DR_, *κ*_M_ = *N*_c*M*_/*T*_DM_, and *κ*_S_ = *N*_c*S*_/*T*_DS_, where the *T*_DR_, *T*_DM_, and *T*_DS_ are the durations of the respective RBC stages, and *N*_c*R*_, *N*_c*M*_, and *N*_c*S*_ are the respective numbers of compartments used for each stage (as set by Equation 2 above). ES(*t*) is the rate of new RBC production at time *t*. Based on physiologically reasonable values [57], we took *T*_DR_ = 36 h with *σ* = 6 h, *T*_DM_ = 2,796 h with *σ* = 168 h, and *T*_DS_ = 48 h with σ = 12 h. For *P. vivax* infections, the model for RBC development is exactly the same, except that the mature and senescent stages are not attacked at all by merozoites. The total uninfected RBC count, *E*_T_, is equal to the sum of all the *R*, *M*, and *S* compartments. If *E*_T_ drops below 3×10^6^ RBCs µl^−1^ (i.e., 0.6×the basal count of a typical healthy adult), then we assume that the host dies of catastrophic anemia. Studies of RBC or hemoglobin levels in patients with either *P. falciparum* [29],[62] or *P vivax* [3] infections suggest that RBC counts can collapse to similarly low fractions of the basal count.

### Erythropoietic Response

The dynamics for the marrow RBC source depend on the host response to losses of uninfected RBCs. Let Φ = ES_0_−d*E*_T_/d*t*−*δζμV*, where ES_0_ is the basal rate of RBC production (which maintains a healthy basal count of 5×10^6^ µl^−1^), and *ζ*, *μ*, and *V* are as above in Equations 3 and 4. The factor *δ* is a simplistic way to account for the dyserythropoietic effects during infection. We model the dynamics of ES(*t*) with the following ODE:
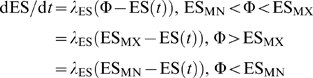
(8)


ES_MX_ is the maximum RBC production rate, and 1/*λ*_ES_ is a response time to changes in the rate of RBC loss. For a healthy compensatory response to RBC loss [57], ES_MX_/ES_0_ = 5 and 1/*λ*_ES_ = 48 h. ES_MN_ is a minimum production rate; with dyserythropoiesis, the RBC rate production would be driven to ES_MN_. As mentioned above, we considered four model RBC source dynamics, the parameters specified in Table 2.

**Table 2 pcbi-1000149-t002:** Values of Parameters for Model Erythropoietic Responses.

Name	1/*λ*_ES_ (h)	*δ*	ES_MX_/ES_0_	ES_MN_/ES_0_
Fixed	Infinite	–	–	–
Compensatory	48	0	5	0.65
Moderate dyserythropoietic	48	4.5	5	0.75
Severe dyserythropoietic	48	8.5	5	0.65

### Immune Response Dynamics

As noted above, we consider two main types of immune actions: a fast-activating but short-acting innate response, and a slower-activating but long-acting antibody-like response. We consider the innate response first. Let *Act* be the actuator level, *Att* the attacker level, and *A* an antigen (targeted parasite stage) that triggers the response. Actuator production above its basal rate is triggered when the density of a target parasite stage *A* exceeds a detection threshold *A*_th_:
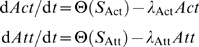
(9)where Θ(*x*) = *x* if *x*>0, zero otherwise, and
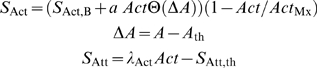



*S*_Act,B_ is the basal actuator level of production, *a* the self-amplification, and *Act*_Mx_ the maximum allowed actuator level. Parameter *S*_Att,th_ is fixed by the other parameters so that production of the attacker does not begin unless the actuator production is above its basal level. Since schizont rupture is believed to trigger the short-term innate reaction associated with paroxysms in *P. vivax* infection [7], we took the actuator phase to be sensitive to the count of IBCs in the last 4% of their development cycle (“bursting schizonts”). Clinical studies have shown rapid growth and quick decay of TNF-α levels in *P. vivax* infections [6], on the order of 1–2 h. Fever spikes follow the TNF-α peaks and also decay quickly in such patients, on the order of 2–3 h. For the simulations reported here, we assumed 1/*λ*_Act_ = 1 h, *Act*_Mx_ = 2.5 µl^−1^, with *S*_Act,B_ set so that the basal actuator level is 0.1, *a* = 100, and 1/*λ*_Att_ = 2 h. Test simulations indicated that model results were rather insensitive to values of these parameters as long as *λ*_Act_ and *λ*_Att_ were ∼1 h^−1^ (results not shown).

Simulation outcomes were sensitive to the antigen detection threshold *A*_th_ (see Equation 9) and to *ξ*, the binding affinity to the target of the response (as defined in Equations 3 and 4). In our simulations, we considered three values of *A*_th_ (given in Table 3). We also considered three targets for this response: (1) IBCs <24 h old, (early trophozoite), (2) IBCs between 24 and 48 h old (late trophozoite and schizont), and (3) IBCs of any age. For each target and sensitivity, we considered three values of binding affinity *ξ* (given in Table 3). In a sense, the product *ξ* *Att* is the tangible quantity, as this is the actual clearance rate of the subpopulation targeted by this response.

**Table 3 pcbi-1000149-t003:** Parameter Values for Model Innate Responses.

Target	*A*_th_ (µl^−1^)	*ξ* (µl h^−1^)
All IBCs	0.5, 5, or 50	0.0008, 0.008, or 0.08
Early trophozoites	0.5, 5, or 50	0.0008, 0.008, or 0.08
Late trophozoites and schizonts	0.5, 5, or 50	0.0008, 0.008, or 0.08
No target	–	0

For the antibody-like response (Ab response), the actuator triggers an intermediate growth phase which imposes a delay on the production of the attacker; thus, we use a multicompartment model for it. Let *G_n_* be the amount of intermediate phase in compartment *n*, and let *T*_DG_ be the duration of the growth phase with standard deviation *S*_DG_:
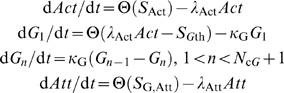
(10)where
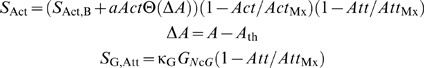



Parameters *S*_Act,B_, *a*, *Act*_Mx_, *λ*_Act_, *A*_th_ and *λ*_Att_ have the same roles as for the innate response, *N*_c*G*_ is set by Equation 2 above, κ_G_ = *NcG/T_DG_*, and *Att*_MX_ is the maximum allowed level of the attacker. S_Gth_ is set so that there is no production of the growth phase unless the actuator is above its basal level. Note, too, that the definition for *S*_Act_ is slightly different from the one in equation 9, as we allow for a shutdown of actuator production once the attacker reaches its maximum level: all production of the attacker shuts down once the attacker level reaches *Att*_Mx_. Results with mouse models suggest that the first specific activated B or T cells appear 4–7 days after inoculation with blood-stage parasites [63],[64], so we take *T*_DG_ = 96 hours, with *σ* = 9.6 hours. As for the innate response, we assume 1/*λ*_Act_ = 1 h, *Act*_Mx_ = 2.5 µl^−1^ and *S*_Act,B_ set so that the basal actuator level is 0.1. However, we took the self-amplification factor *a* to be much smaller, 0.01. We set *Att*_Mx_ = 10 µl^−1^. Again, the product *ξAtt* is the tangible quantity, not *Att* or *ξ* directly.

For the Ab response, we assume that the targeted subpopulations of parasites also provide the trigger for the actuator. We considered five possible targets: (1) early trophozoite, (2) late trophozoite and schizont, (3) bursting schizonts (as defined above), (4) IBCs of any age, and (5) merozoites. Test simulations indicated that if merozoites were the target of the model antibody response, then the binding affinity *ξ* had to be many orders of magnitude larger and the trigger threshold *A*_th_ much smaller than if the other subpopulations were the target in order to achieve the same degree of control on parasitemia. (This is in line with other studies [13],[34]; see Results section above.) The values used for *A*_th_ and *ξ* for each of the targeted parasite populations are given in Table 4. For all choices of target population, *A*_th_ and *ξ*, we considered three values for the time decay constant of the attacker phase, *λ*_Att_ (also listed in Table 4). For simplicity, we do not directly consider the effects of antigenic variation in *P. falciparum* infection. However, in order to consider the effects of an ineffective adaptive response, we did simulations with an innate response but no antibody response, and, symmetrically, simulations with an antibody response but no innate response.

**Table 4:** Values of Parameters for Model Antibody Responses.

Target	*A*_th_ (µl^−1^)	*ξ* (µl h^−1^)	1/*λ*_Att_ (h)
All IBCs	0.5, 5	0.0008, 0.008, or 0.08	160, 720, or 8760
Early trophozoites	0.5, 5	0.0008, 0.008, or 0.08	160, 720, or 8760
Late trophozoites and schizonts	0.5, 5	0.0008, 0.008, or 0.08	160, 720, or 8760
“Bursting” schizonts	0.5, 5	0.0008, 0.008, or 0.08	160, 720, or 8760
Merozoites	0.01	10, 100, 1000 or 10000	160, 720, or 8760
No target	–	0	–

For most of the simulations, we started with an antibody-naïve host; i.e., the initial level of attacker component is zero. However, for all model innate and erythropoietic responses to *P. vivax* infection, we did addition simulations with pre-existing antibodies of the response that targets IBCs of any age. The parameters *ξ*, *A*_th_, and *λ*_Att_ used for this set of simulations have the same values as those listed for the “All IBCs” model antibody response in Table 4. As mentioned above, the initial antibody level is set at *e* ^−1^×*Att*_Mx_, or ∼3.679 µl^−1^.

### Simulation Strategy

Tables 1–4 summarize the values used for all of the model parameters which we varied from simulation to simulation. A simulation was done for each possible choice of parameters in the tables, thus results of 38,080 simulations for antibody-naïve hosts are reported for each of the two *Plasmodium* species. In addition, we did a further 8,064 simulations of *P. vivax* infection with pre-existing antibodies. Model infections were simulated for up to one year after the primary release of parasites from the liver into the bloodstream, unless the host died from catastrophic anemia or the parasite was cleared from the host before a full year elapsed. Although the ODE system has hundreds to thousands of dependent compartmental variables to solve, it is not stiff in the parameter regimes of interest, and thus can be solved efficiently. Of course, we still only examined a small part of the parameter space of the model, and in many ways our model is a caricature of the real parasite-host system. Nonetheless, in sweeping across these many combinations of parameter values we identified several basic, broad trends with changes in the structures of the immune and erythropoietic responses.

If at any point the merozoite count, the total infected RBC count, or the total uninfected RBC count dropped to <1 in a total blood volume of 5×10^6^ µl, the values of all compartments that contributed to that particular count were reset to zero. The CODE system was solved using the fifth-order Runge-Kutta-Fehlberg algorithm with adaptive stepsize control for time integration [65],[66], so that the difference between the fourth- and fifth-order solutions for each component of the ODE systems was less than one part in 10^6^.

## Supporting Information

Figure S1 Plots illustrating results for certain subsets of simulated infections. (A) Peak parasitemia *I*_PK_ versus integrated parasitemia *I*_INT_ for all model *P. falciparum* infections with fixed RBC rate of production and antibody naïve host. (B) RBC deficit ΔRBC versus integrated parasitemia *I*_INT_ for all model *P. falciparum* infections with antibody naïve hosts and with fixed RBC rate of production (black points) and or with moderate dyserythropoiesis (gray points). (C) Peak parasitemia *I*_PK_ versus integrated parasitemia I_INT_ for all model *P. vivax* infections with fixed RBC rate of production with pre-existing antibodies that attack all IBCs. (D) RBC deficit ΔRBC versus integrated parasitemia *I*_INT_ for all model *P. vivax* infections with pre-existing antibodies that attack all IBCs and with fixed RBC rate of production (black points) and or with moderate dyserythropoiesis (gray points).
(0.88 MB TIF)

Figure S2 Overall variation in variation in anemia with different combinations of *Plasmodium* species, erythropoietic, antibody and intraerythrocytic development time (*σ*_IBC_) for model infections in antibody naïve hosts with no innate response. (A) ΔRBC averaged over all simulations with the given combination of species, erythropoietic response, and antibody target. (B) ΔRBC averaged over all simulations with the given combination of species, *σ*_IBC_, and antibody target. (C) Color code for the lines in panels (A) and (B). Abbreviations for antibody and erythropoietic responses as in Figure 4 of text. If the data points and connecting lines for two or more antibody responses overlap in the plot, the lines are dashed to reveal all the responses present.
(0.25 MB TIF)

Figure S3 Variation in time until resolution of infection with different combinations of *Plasmodium* species, erythropoietic and antibody responses for infections in antibody naïve hosts without innate responses. (A) Time after primary release until death averaged over all those infections which ended in death of host by anemia, and (B) time from primary release until clearance of parasite from host averaged over all those infections in which the host cleared the parasite within one year for the given combination of species, erythropoietic response, and antibody target. (C) Color code for the data points and lines. Abbreviations as in Figure 4 in the main text. Lines are just to guide the eye. If the data points and connecting lines for two or more antibody responses overlap in the plot, the lines are dashed to reveal all the responses present. Note: there was no clearance of infections in model host that either (i) lack an innate response and an antibody response or (ii) lack an innate response but had an antibody response to bursting schizonts.
(0.20 MB TIF)

Figure S4 Overall variation in outcome for different erythropoietic responses to *P. vivax* infections in hosts with pre-existing antibodies to IBCs of any stage but with no innate immunity. (A) *I*_PK_, (B) *I*_INT_ and (C) ΔRBC averaged over all simulations with the given response, (D) fraction of simulations with a given response in which the host died, (E) fraction of simulations with a given response in which the parasite was cleared. Abbreviations as in Figure 4 in the main text. Lines are just to guide the eye.
(0.20 MB TIF)

Figure S5 Overall variation in variation in anemia with different combinations of *Plasmodium* species, erythropoietic, antibody and innate responses for model infections in antibody naïve hosts with an innate response. (A) ΔRBC averaged over all simulations with the given combination of species, erythropoietic response, and antibody target. (B) Color code for the lines in panel (A). Abbreviations for antibody responses as in Figure 4 of text. (C) ΔRBC averaged over all simulations with the given combination of species, erythropoietic response, and innate target. (D) Color code for the lines in panel (C). Abbreviations for innate responses as in Figure 7 of text. If the data points and connecting lines for two or more antibody responses overlap in the plot, the lines are dashed to reveal all the responses present.
(0.41 MB TIF)

Figure S6 Variation in time until resolution of infection with different combinations of *Plasmodium* species, erythropoietic and antibody responses for model infections in antibody naïve hosts that mount an innate responses. (A) Time after primary release until death averaged over all those infections which ended in death of host by anemia, and (B) time from primary release until clearance of parasite from host averaged over all those infections in which the host cleared the parasite within one year for the given combination of species, erythropoietic response, and antibody target. (C) Color code for the data points and lines. Abbreviations as in Figure 4 in the main text. Lines are just to guide the eye. If the data points and connecting lines for two or more antibody responses overlap in the plot, the lines are dashed to reveal all the responses present. Note: no host with *P. vivax* infection died unless there was dyserthropoiesis. No host with *P. falciparum* infection died unless there was severe dyserthropoiesis.
(0.23 MB TIF)

Figure S7 Overall variation in outcome for different combinations of innate and erythropoietic responses to *P. vivax* infections in hosts with pre-existing antibodies to IBCs of any stage and that mount an innate immunity. (A) *I*_PK_, (B) *I*_INT_ and (C) ΔRBC averaged over all simulations with the given combination of responses, (D) fraction of simulations with a given combination in which the host died, (E) fraction of simulations with a given combination in which the parasite was cleared. Abbreviations as in Figure 7 in text. Lines are just to guide the eye.
(0.27 MB TIF)

Figure S8 Overall variation in variation in anemia with different combinations of *Plasmodium* species, standard deviations in the intraerythrocytic development time (*σ*_IBC_), antibody and innate responses for model infections in antibody naïve hosts with an innate response. (A) ΔRBC averaged over all simulations with the given combination of species, *σ*_IBC_, and antibody target. (B) Color code for the lines in panel (A). Abbreviations for antibody responses as in Figure 4 of text. (C) ΔRBC averaged over all simulations with the given combination of species, σ_IBC_, and innate target. (D) Color code for the lines in panel (C). Abbreviations for innate responses as in Figure 7 of text. If the data points and connecting lines for two or more antibody responses overlap in the plot, the lines are dashed to reveal all the responses present.
(0.30 MB TIF)

Figure S9 Variation in time until resolution of infection with different combinations of *Plasmodium* species, standard deviations in the intraerythrocytic development time (*σ*_IBC_), and antibody responses for model infections in antibody naïve hosts that mount an innate responses. (A) Time after primary release until death averaged over all those infections which ended in death of host by anemia and (B) time from primary release until clearance of parasite from host averaged over all those infections in which the host cleared the parasite within one year for the given combination of species, *σ*_IBC_, and antibody target. (C) Color code for the data points and lines. Abbreviations as in Figure 4 in the main text. Lines are just to guide the eye. If the data points and connecting lines for two or more antibody responses overlap in the plot, the lines are dashed to reveal all the responses present. Note: no host with either (i) antibodies to bursting schizonts, (ii) merozoites, or (iii) lacking antibodies could clear the parasite unless *σ*_IBC_<4.8 hours.
(0.27 MB TIF)

Figure S10 Overall variation in outcome for different combinations of standard deviations in the intraerythrocytic development time (*σ*_IBC_) and innate responses to *P. vivax* infections in hosts with pre-existing antibodies to IBCs of all stages and that mount an innate immunity. (A) *I*_PK_, (B) *I*_INT_ and (C) ΔRBC averaged over all simulations with the given combination of responses, (D) fraction of simulations with a given combination in which the host died, (E) fraction of simulations with a given combination in which the parasite was cleared. Abbreviations as in Figure 7 in text. Lines are just to guide the eye.
(0.28 MB TIF)
