# Supplementary figures and images for: Host Control of Malaria Infections: Constraints on Immune and Erythropoeitic Response Kinetics
Source: PLoS Comput Biol. 2008 Aug 22;4(8):e1000149. doi: 10.1371/journal.pcbi.1000149 (PMC2491590; doi:10.1371/journal.pcbi.1000149)

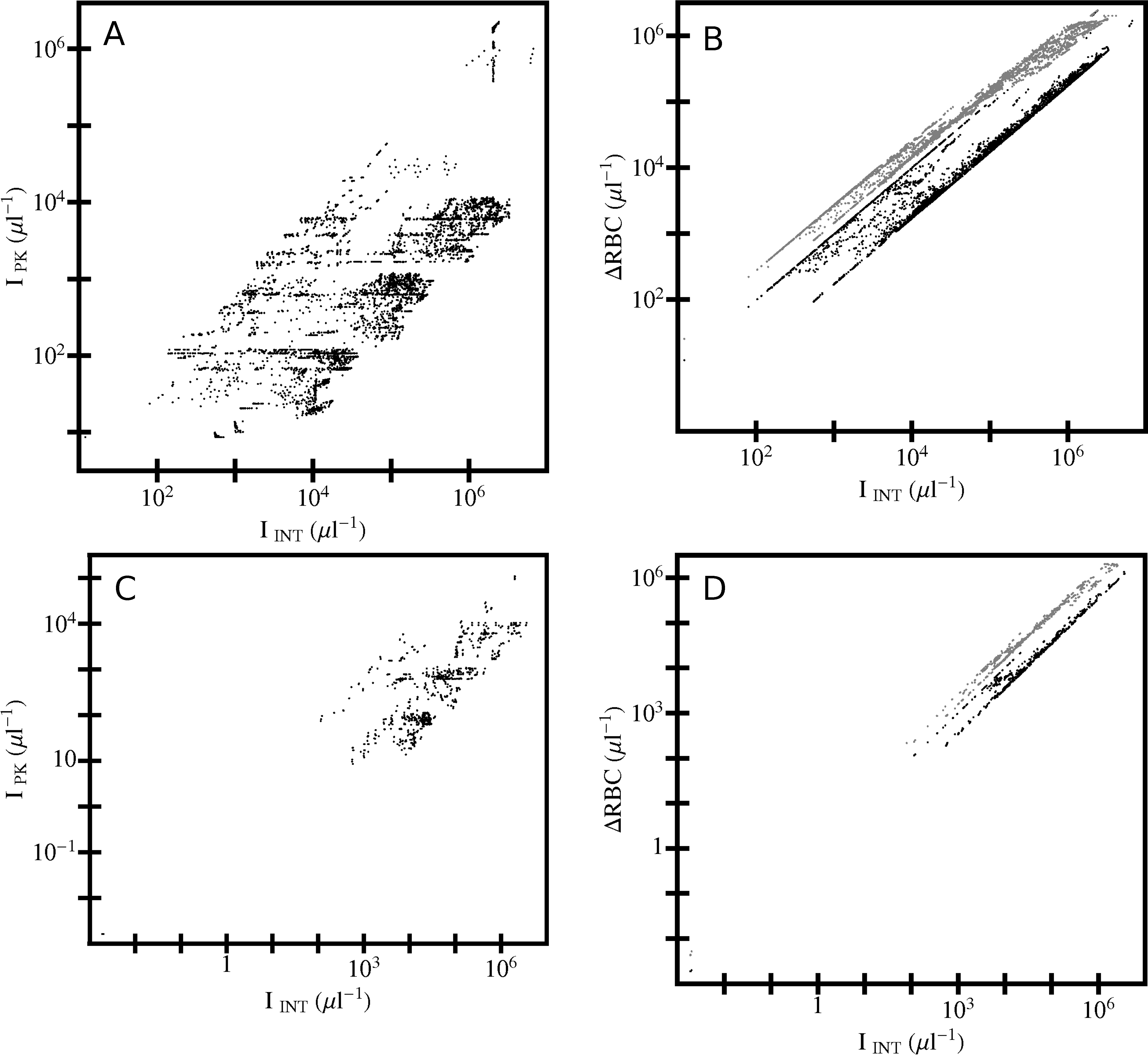

Supplement: Figure S1 — Plots illustrating results for certain subsets of simulated infections. (A) Peak parasitemia I PK versus integrated parasitemia I INT for all model P. falciparum infections with fixed RBC rate of production and antibody naïve host. (B) RBC deficit ΔRBC versus integrated parasitemia I INT for all model P. falciparum infections with antibody naïve hosts and with fixed RBC rate of production (black points) and or with moderate dyserythropoiesis (gray points). (C) Peak parasitemia I PK versus integrated parasitemia IINT for all model P. vivax infections with fixed RBC rate of production with pre-existing antibodies that attack all IBCs. (D) RBC deficit ΔRBC versus integrated parasitemia I INT for all model P. vivax infections with pre-existing antibodies that attack all IBCs and with fixed RBC rate of production (black points) and or with moderate dyserythropoiesis (gray points). (0.88 MB TIF) [file pcbi.1000149.s001.tif]

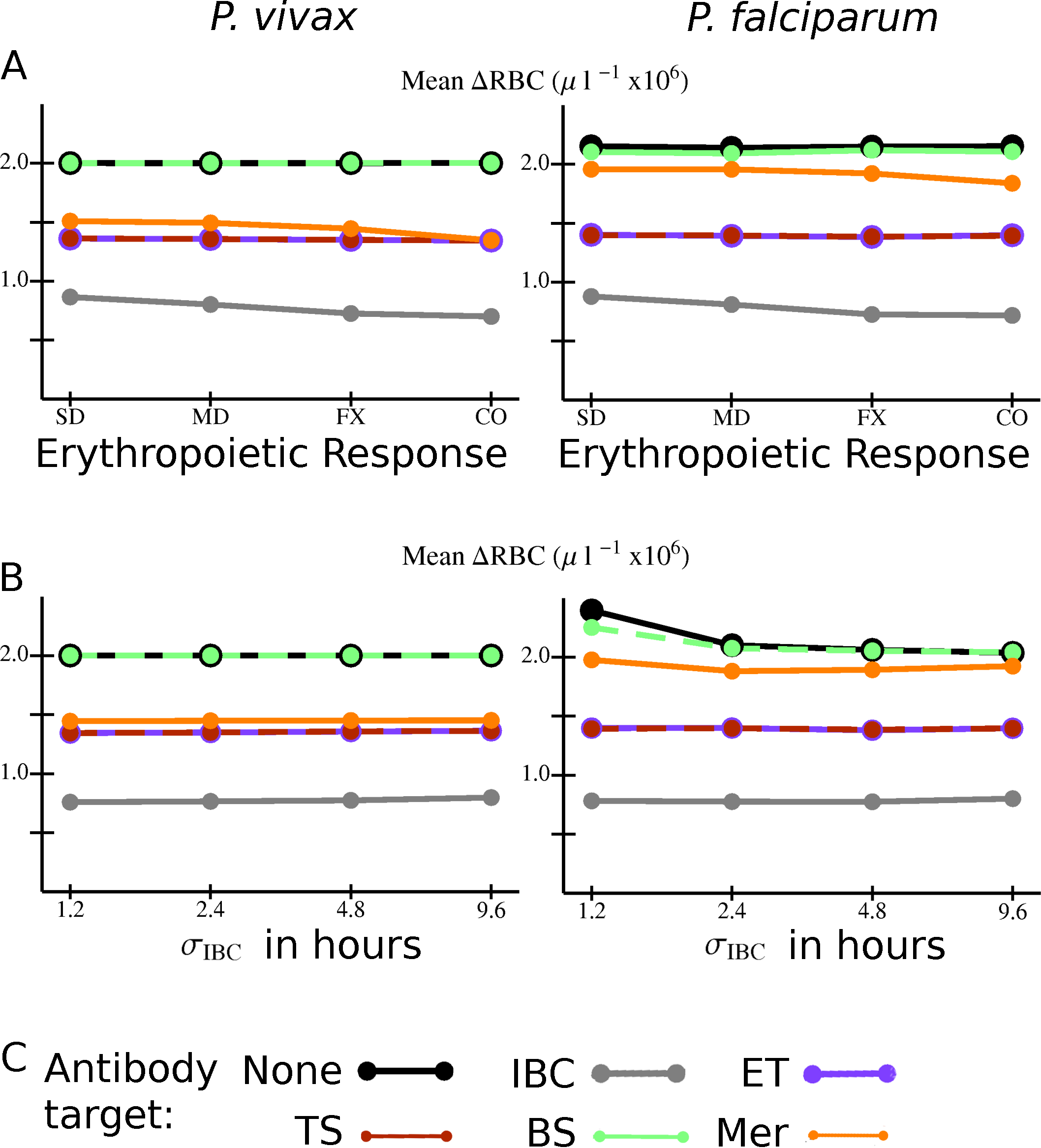

Supplement: Figure S2 — Overall variation in variation in anemia with different combinations of Plasmodium species, erythropoietic, antibody and intraerythrocytic development time (σ IBC) for model infections in antibody naïve hosts with no innate response. (A) ΔRBC averaged over all simulations with the given combination of species, erythropoietic response, and antibody target. (B) ΔRBC averaged over all simulations with the given combination of species, σ IBC, and antibody target. (C) Color code for the lines in panels (A) and (B). Abbreviations for antibody and erythropoietic responses as in Figure 4 of text. If the data points and connecting lines for two or more antibody responses overlap in the plot, the lines are dashed to reveal all the responses present. (0.25 MB TIF) [file pcbi.1000149.s002.tif]

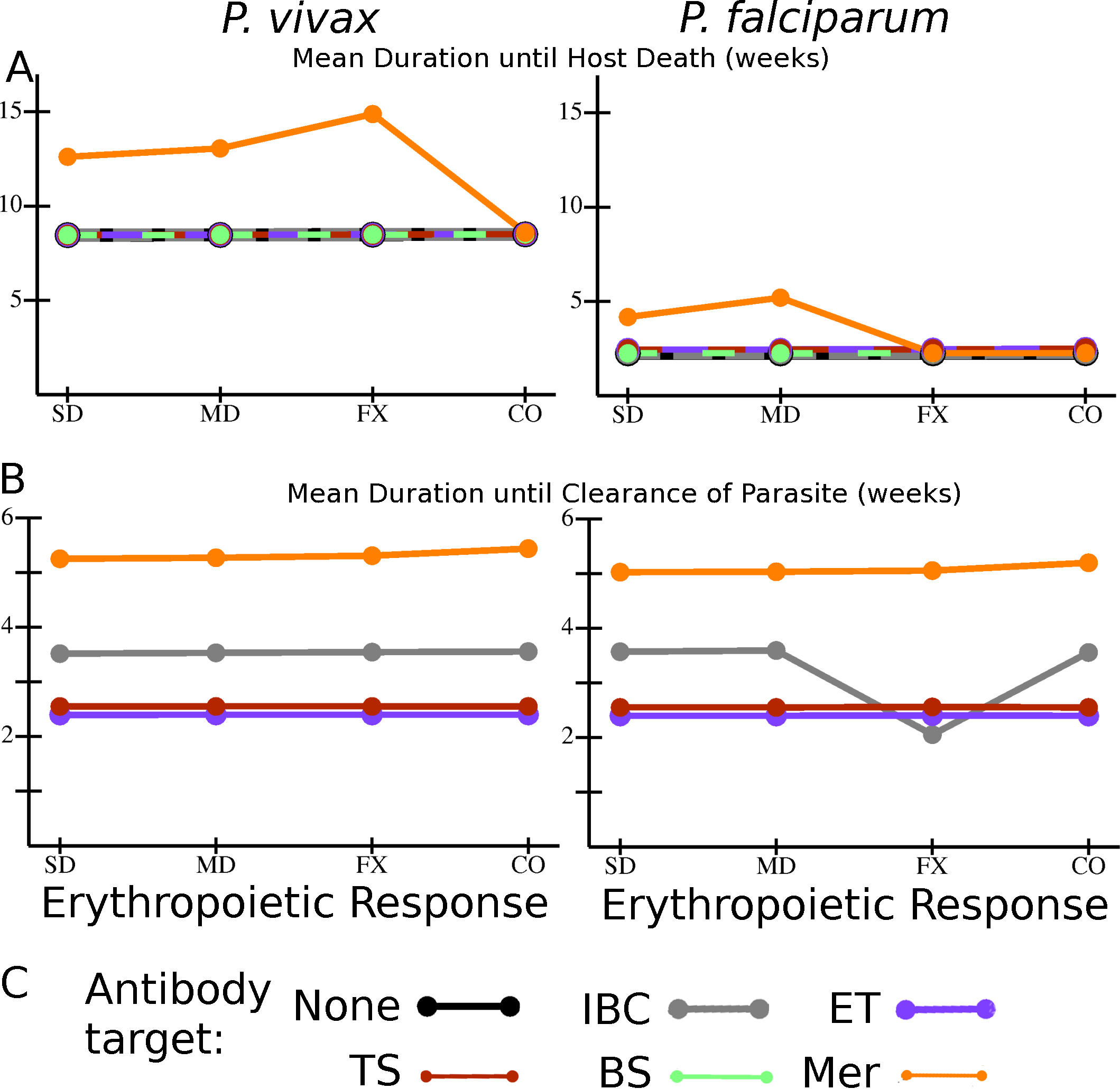

Supplement: Figure S3 — Variation in time until resolution of infection with different combinations of Plasmodium species, erythropoietic and antibody responses for infections in antibody naïve hosts without innate responses. (A) Time after primary release until death averaged over all those infections which ended in death of host by anemia, and (B) time from primary release until clearance of parasite from host averaged over all those infections in which the host cleared the parasite within one year for the given combination of species, erythropoietic response, and antibody target. (C) Color code for the data points and lines. Abbreviations as in Figure 4 in the main text. Lines are just to guide the eye. If the data points and connecting lines for two or more antibody responses overlap in the plot, the lines are dashed to reveal all the responses present. Note: there was no clearance of infections in model host that either (i) lack an innate response and an antibody response or (ii) lack an innate response but had an antibody response to bursting schizonts. (0.20 MB TIF) [file pcbi.1000149.s003.tif]

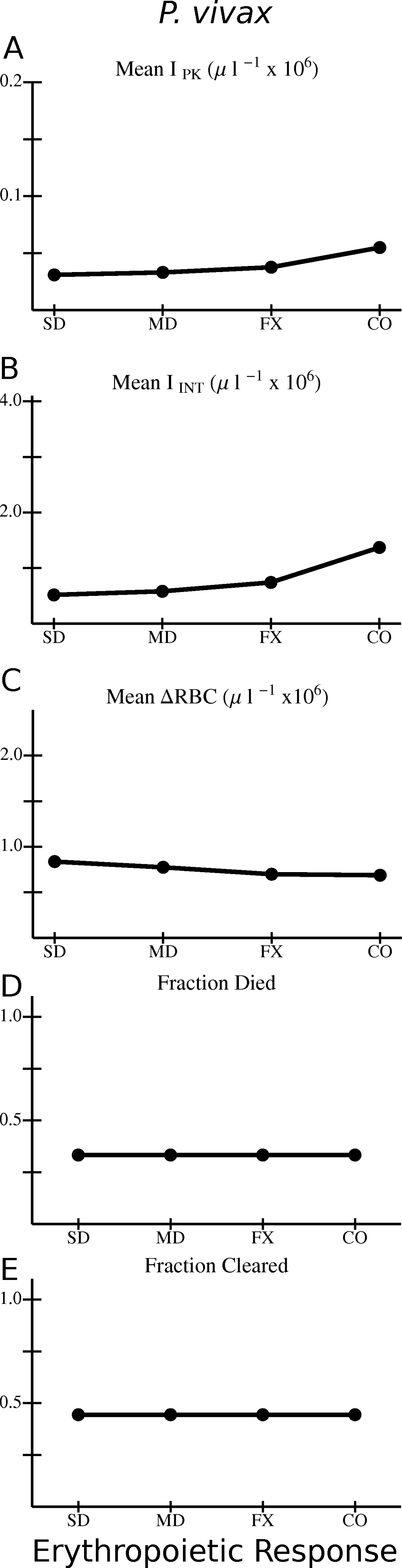

Supplement: Figure S4 — Overall variation in outcome for different erythropoietic responses to P. vivax infections in hosts with pre-existing antibodies to IBCs of any stage but with no innate immunity. (A) I PK, (B) I INT and (C) ΔRBC averaged over all simulations with the given response, (D) fraction of simulations with a given response in which the host died, (E) fraction of simulations with a given response in which the parasite was cleared. Abbreviations as in Figure 4 in the main text. Lines are just to guide the eye. (0.20 MB TIF) [file pcbi.1000149.s004.tif]

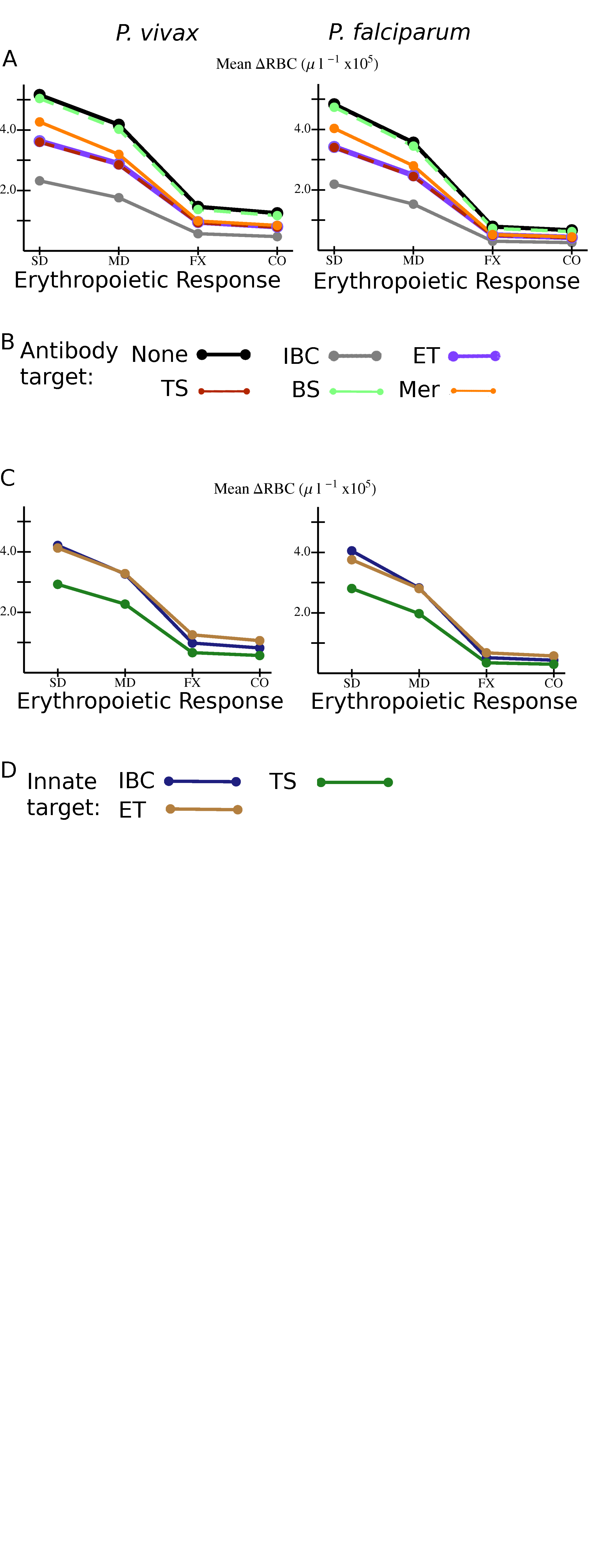

Supplement: Figure S5 — Overall variation in variation in anemia with different combinations of Plasmodium species, erythropoietic, antibody and innate responses for model infections in antibody naïve hosts with an innate response. (A) ΔRBC averaged over all simulations with the given combination of species, erythropoietic response, and antibody target. (B) Color code for the lines in panel (A). Abbreviations for antibody responses as in Figure 4 of text. (C) ΔRBC averaged over all simulations with the given combination of species, erythropoietic response, and innate target. (D) Color code for the lines in panel (C). Abbreviations for innate responses as in Figure 7 of text. If the data points and connecting lines for two or more antibody responses overlap in the plot, the lines are dashed to reveal all the responses present. (0.41 MB TIF) [file pcbi.1000149.s005.tif]

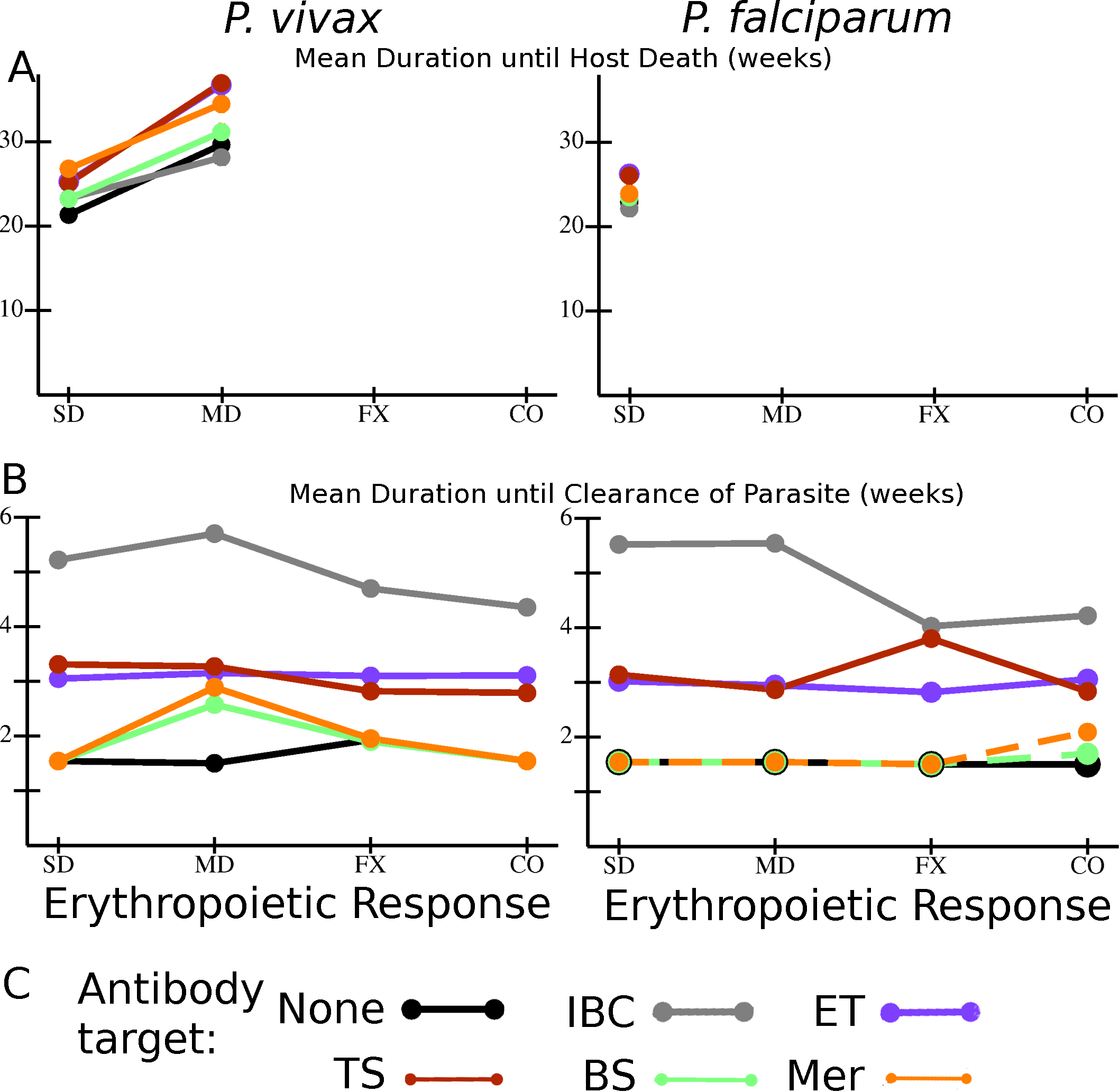

Supplement: Figure S6 — Variation in time until resolution of infection with different combinations of Plasmodium species, erythropoietic and antibody responses for model infections in antibody naïve hosts that mount an innate responses. (A) Time after primary release until death averaged over all those infections which ended in death of host by anemia, and (B) time from primary release until clearance of parasite from host averaged over all those infections in which the host cleared the parasite within one year for the given combination of species, erythropoietic response, and antibody target. (C) Color code for the data points and lines. Abbreviations as in Figure 4 in the main text. Lines are just to guide the eye. If the data points and connecting lines for two or more antibody responses overlap in the plot, the lines are dashed to reveal all the responses present. Note: no host with P. vivax infection died unless there was dyserthropoiesis. No host with P. falciparum infection died unless there was severe dyserthropoiesis. (0.23 MB TIF) [file pcbi.1000149.s006.tif]

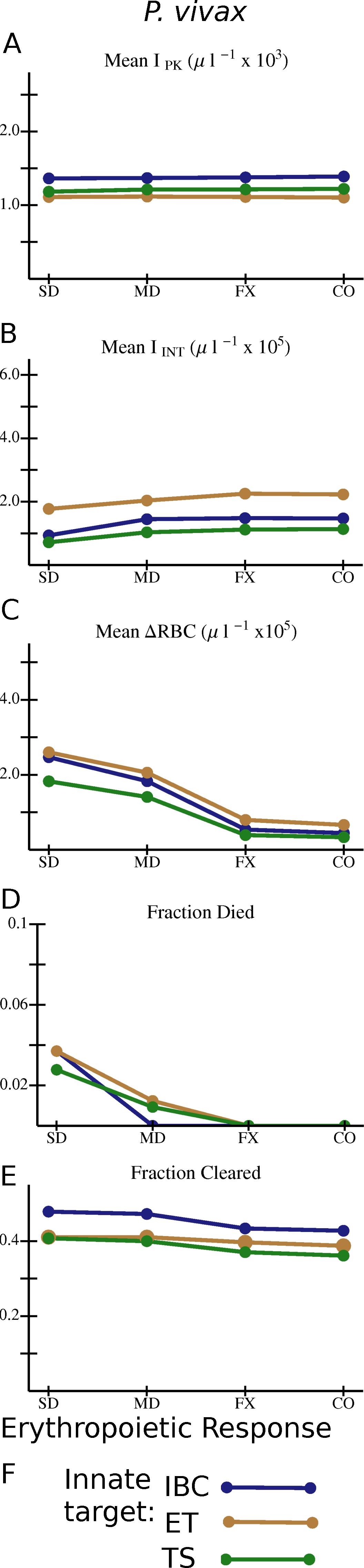

Supplement: Figure S7 — Overall variation in outcome for different combinations of innate and erythropoietic responses to P. vivax infections in hosts with pre-existing antibodies to IBCs of any stage and that mount an innate immunity. (A) I PK, (B) I INT and (C) ΔRBC averaged over all simulations with the given combination of responses, (D) fraction of simulations with a given combination in which the host died, (E) fraction of simulations with a given combination in which the parasite was cleared. Abbreviations as in Figure 7 in text. Lines are just to guide the eye. (0.27 MB TIF) [file pcbi.1000149.s007.tif]

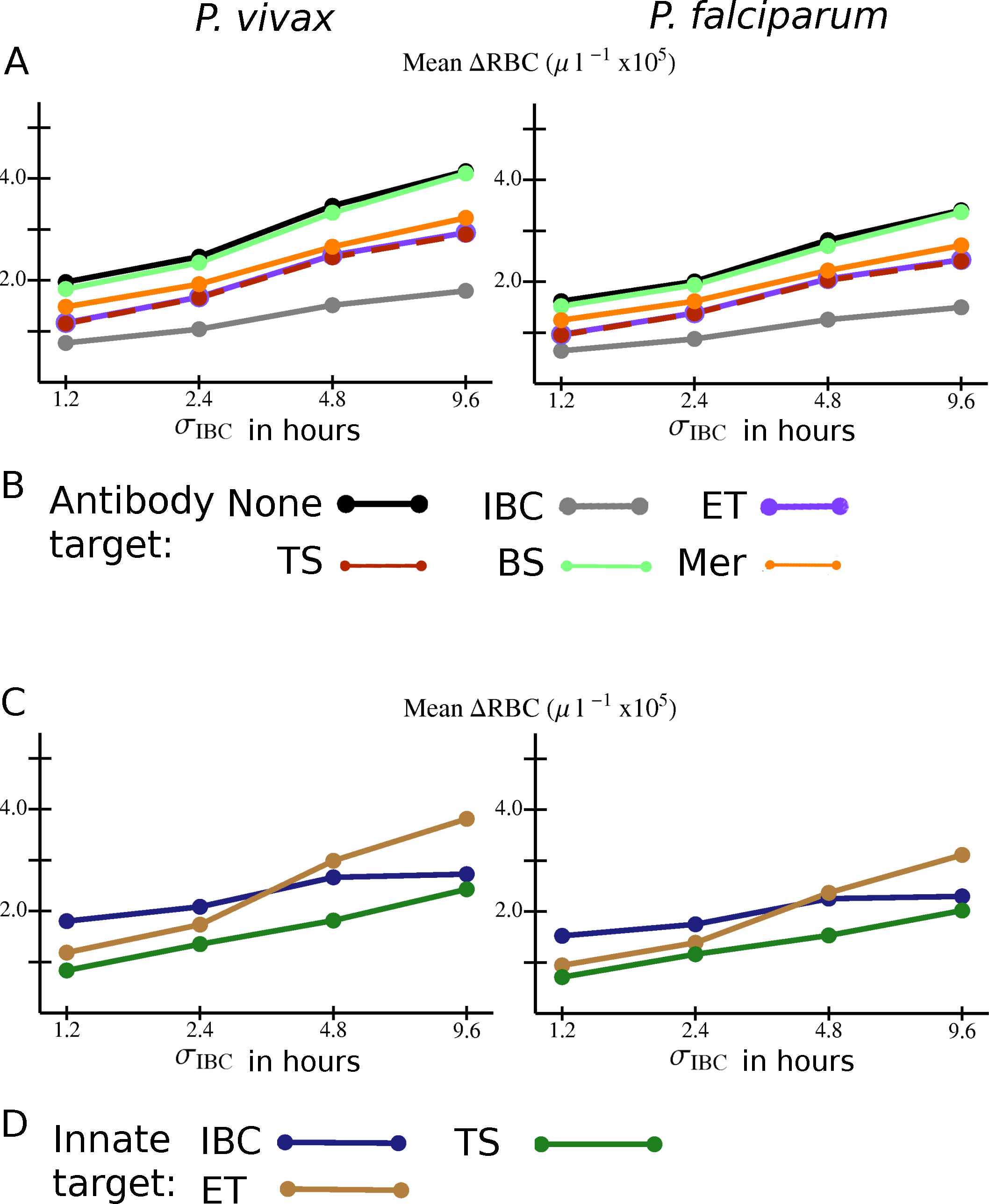

Supplement: Figure S8 — Overall variation in variation in anemia with different combinations of Plasmodium species, standard deviations in the intraerythrocytic development time (σ IBC), antibody and innate responses for model infections in antibody naïve hosts with an innate response. (A) ΔRBC averaged over all simulations with the given combination of species, σ IBC, and antibody target. (B) Color code for the lines in panel (A). Abbreviations for antibody responses as in Figure 4 of text. (C) ΔRBC averaged over all simulations with the given combination of species, σIBC, and innate target. (D) Color code for the lines in panel (C). Abbreviations for innate responses as in Figure 7 of text. If the data points and connecting lines for two or more antibody responses overlap in the plot, the lines are dashed to reveal all the responses present. (0.30 MB TIF) [file pcbi.1000149.s008.tif]

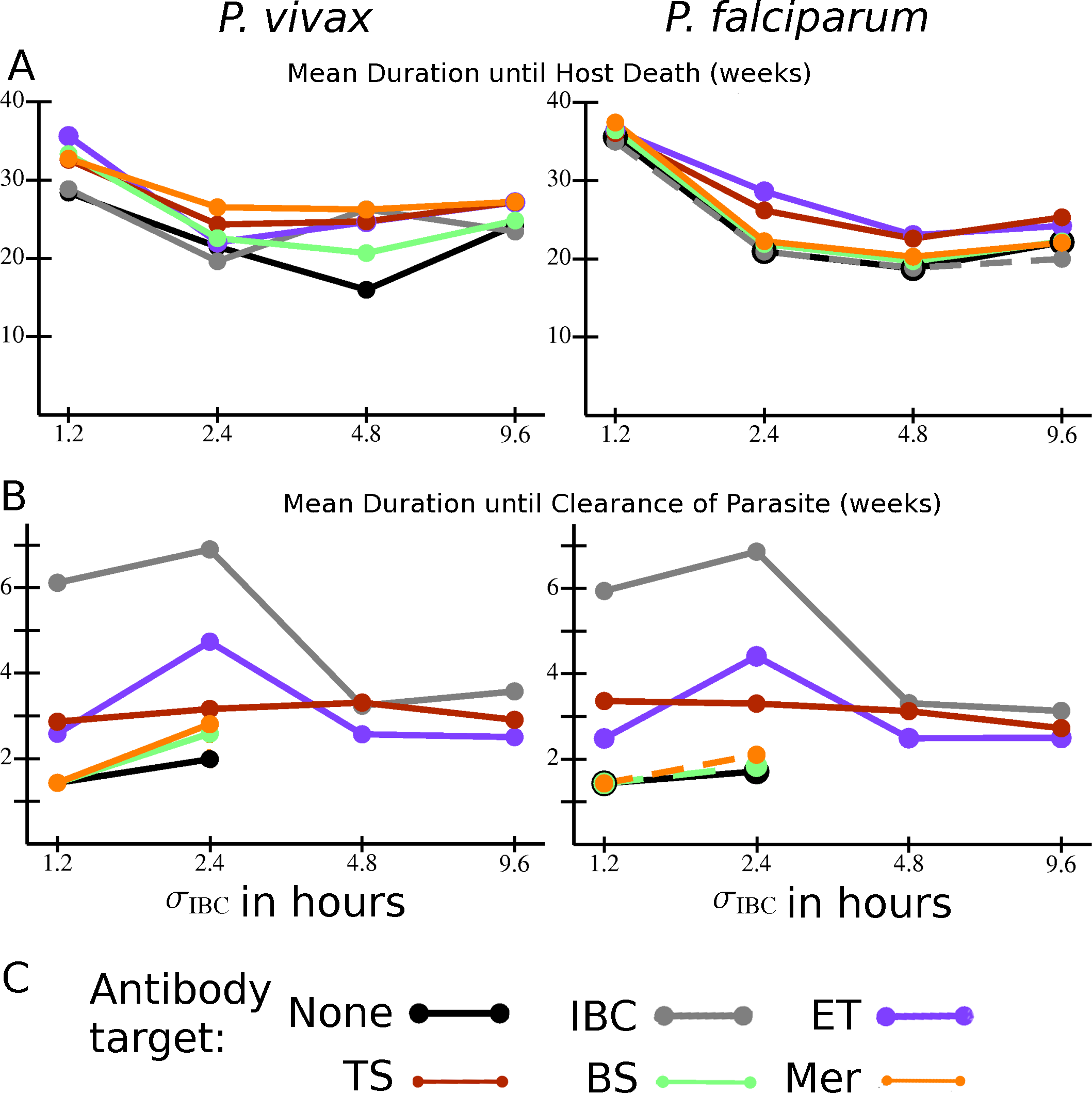

Supplement: Figure S9 — Variation in time until resolution of infection with different combinations of Plasmodium species, standard deviations in the intraerythrocytic development time (σ IBC), and antibody responses for model infections in antibody naïve hosts that mount an innate responses. (A) Time after primary release until death averaged over all those infections which ended in death of host by anemia and (B) time from primary release until clearance of parasite from host averaged over all those infections in which the host cleared the parasite within one year for the given combination of species, σ IBC, and antibody target. (C) Color code for the data points and lines. Abbreviations as in Figure 4 in the main text. Lines are just to guide the eye. If the data points and connecting lines for two or more antibody responses overlap in the plot, the lines are dashed to reveal all the responses present. Note: no host with either (i) antibodies to bursting schizonts, (ii) merozoites, or (iii) lacking antibodies could clear the parasite unless σ IBC<4.8 hours. (0.27 MB TIF) [file pcbi.1000149.s009.tif]

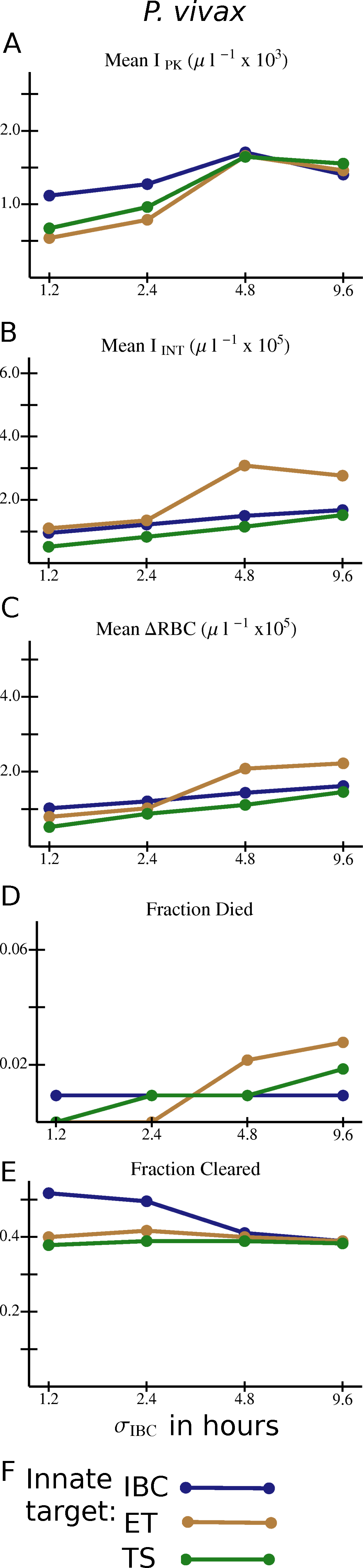

Supplement: Figure S10 — Overall variation in outcome for different combinations of standard deviations in the intraerythrocytic development time (σ IBC) and innate responses to P. vivax infections in hosts with pre-existing antibodies to IBCs of all stages and that mount an innate immunity. (A) I PK, (B) I INT and (C) ΔRBC averaged over all simulations with the given combination of responses, (D) fraction of simulations with a given combination in which the host died, (E) fraction of simulations with a given combination in which the parasite was cleared. Abbreviations as in Figure 7 in text. Lines are just to guide the eye. (0.28 MB TIF) [file pcbi.1000149.s010.tif]
